# Small RNA fragments derived from multiple RNA classes – the missing element of multi-omics characteristics of the hepatitis C virus cell culture model

**DOI:** 10.1186/s12864-017-3891-3

**Published:** 2017-06-30

**Authors:** Paulina Jackowiak, Anna Hojka-Osinska, Anna Philips, Agnieszka Zmienko, Lucyna Budzko, Patrick Maillard, Agata Budkowska, Marek Figlerowicz

**Affiliations:** 1 Institute of Bioorganic Chemistry, Polish Academy of Sciences, Noskowskiego 12/14, 61-704 Poznan, Poland; 2 Institute of Computing Science, Poznan University of Technology, Piotrowo 3A, 60-965 Poznan, Poland; 3 Institut Pasteur, Hepacivirus and Innate Immunity, CNRS, UMR3569, 75724 Paris, France; 4 Scientific Advisor for the Department of International Affairs, Institut Pasteur, 75724 Paris, France

**Keywords:** Non-coding RNA, RNA fragments, tRF, HCV

## Abstract

**Background:**

A pool of small RNA fragments (RFs) derived from diverse cellular RNAs has recently emerged as a rich source of functionally relevant molecules. Although their formation and accumulation has been connected to various stress conditions, the knowledge on RFs produced upon viral infections is very limited. Here, we applied the next generation sequencing (NGS) to characterize RFs generated in the hepatitis C virus (HCV) cell culture model (HCV-permissive Huh-7.5 cell line).

**Results:**

We found that both infected and non-infected cells contained a wide spectrum of RFs derived from virtually all RNA classes. A significant fraction of identified RFs accumulated to similar levels as miRNAs. Our analysis, focused on RFs originating from constitutively expressed non-coding RNAs, revealed three major patterns of parental RNA cleavage. We found that HCV infection induced significant changes in the accumulation of low copy number RFs, while subtly altered the levels of high copy number ones. Finally, the candidate RFs potentially relevant for host-virus interactions were identified.

**Conclusions:**

Our results indicate that RFs should be considered an important component of the Huh-7.5 transcriptome and suggest that the main factors influencing the RF biogenesis are the RNA structure and RNA protection by interacting proteins. The data presented here significantly complement the existing transcriptomic, miRnomic, proteomic and metabolomic characteristics of the HCV cell culture model.

**Electronic supplementary material:**

The online version of this article (doi:10.1186/s12864-017-3891-3) contains supplementary material, which is available to authorized users.

## Background

In recent years, the spectrum of known small non-coding RNAs has significantly expanded along with the discovery that virtually all RNA classes reproducibly give rise to a broad repertoire of stable, well-defined fragments [1–4]. Their accumulation levels depend on the cell type and physiological conditions [2, 5–7]. Such RNA fragments (RFs) have been identified across kingdoms of life, and some of them have been proven to have regulatory functions [1–3, 7, 8]. The most extensive functional studies of RFs currently focus on their role in RNA silencing pathways and the regulation of translation. There are several reports showing that fragments derived from a variety of RNA classes (rRNA, snRNA, tRNA, mRNA and vault RNA) associate with Argonaute (Ago) proteins, the key components of the RNA-induced silencing complex (RISC) [9–11]. However, silencing capacities were proven for only a few of these RFs [3, 12–14]. Moreover, some observations suggest that Ago-binding RFs can modulate the posttranscriptional regulation of gene expression via competition with small interfering RNA (siRNA) and/or micro RNA (miRNA) for RNA silencing machinery proteins [11]. Another well-established role of RFs is the regulation of translation [15–19]. They inhibit protein biosynthesis by interaction with translation initiation factors [16] or with ribosomes [18, 19]. Several other postulated functions of RFs include: (i) tRNase Z guiding [20], (ii) regulation of p53-dependent apoptosis [21], (iii) regulation of alternative splicing [22], and (iv) involvement in signaling pathways in plants [23]. Considering the ever growing catalog of mechanisms that engage small RNAs as regulators [24–26], RFs have emerged as an important component of each cell and a rich source of potentially functional molecules.

Since the discovery of RFs, it has been observed that they are formed in response to various pathological processes. There are a number of reports showing a prominent accumulation of RFs under different stress conditions as well as in cancer [5, 27–30]. Unfortunately, little is currently known regarding RF production in association with viral infections. The production of tRNA-derived fragments (tRFs) was observed in cells infected with human respiratory syncytial virus (RSV). One of these tRFs, tRF5Glu-CTC, suppressed host apolipoprotein E receptor 2 (APOER2) mRNA and thus promoted RSV replication [31, 32]. In contrast, increased accumulation of tRFs was not observed in cells infected with human metapneumovirus (hMPV). In case of hMPV infection, tRF profiles did not change despite considerable alterations in the miRNA pool [33]. Significant changes in the accumulation levels of several tRFs were also reported upon apple stem grooving virus (ASGV) infection. The authors of this report suggested that tRFs might be involved in a host-virus interplay [34]. Recently, Selitsky and coworkers reported on RF accumulation in the liver cells of patients with advanced chronic hepatitis B or C and associated hepatocellular carcinoma (HCC). They found that the levels of tRNA-halves were significantly increased in non-malignant liver tissue of patients with chronic viral infections [35]. The tRNA-halves accumulated at lower levels in HCC tissue and were least abundant in the FT3–7 cell line, which is a clonal derivative of Huh-7 cells (a well-differentiated hepatocyte-derived cellular carcinoma cell line) obtained following transformation with a Toll-like receptor 3 (TLR3) expression vector. It is worth noting that little is currently known regarding fragments that form upon viral infections and are derived from classes other than tRNA.

Globally, the hepatitis C virus (HCV) is a leading cause of persistent liver infections that can result in cirrhosis and hepatocellular carcinoma. HCV has a single-stranded (+)RNA genome, in which a single open reading frame is flanked by regulatory 5′ and 3′ untranslated regions (5′ and 3′ UTRs) [36]. The RNA character of the HCV genome has significant implications for viral replication cycle and host-virus interactions. Firstly, the genome is copied by an error-prone RNA-dependent RNA polymerase, which results in the generation of a set of diverse variants, referred to as quasispecies [37–39]. This high genetic variability allows the virus to rapidly adapt to environmental changes, avoid host immune system response and produce drug-resistant mutants; thus, it is considered a therapeutic challenge [40]. Secondly, HCV infections involve extensive interactions between the viral genome and a variety of host-encoded RNA-binding proteins, as well as a cross-talk with miRNA pathways [41–49].

For the long time our knowledge on factors shaping the early stages of HCV infection had been very limited. The situation changed after the development of the Huh-7.5 cell line-based HCV cell culture (HCVcc) model. Its application has allowed to elucidate key aspects of viral infections and host-virus interactions [50]. Our understanding of acute and chronic hepatitis C has been greatly accelerated by high-throughput analyses of the HCVcc transcriptome, miRNome, proteome and metabolome [51–54]. However, information regarding RFs generated at the very beginning of HCV infection, before chronicity is established, is still missing.

Considering the recent evidence demonstrating the importance of RFs in various cellular processes, we characterized the pool of small RNAs (15–82 nt long) that accumulate in the Huh-7.5 HCVcc model. We found that Huh-7.5 cells contained a broad spectrum of RFs derived from multiple RNA classes. The vast majority of these fragments had well-fixed lengths and were repeatedly generated from the same regions of their parental molecules. This observation strongly suggested that specific cellular mechanisms control the process of RF formation. Consequently, we identified several patterns according to which particular RNAs were cleaved into fragments. The fact that a number of RFs accumulated to levels that were similar to those of the miRNAs suggested that RFs could not be neutral to the cell. We observed HCV infection-induced remodeling of the RF pool in Huh-7.5 cells. The levels of low copy number RFs significantly increased in infected cells and the accumulation of high copy number RFs displayed only subtle changes. Accordingly, one can assume that RFs constitute a considerable component of the cellular landscape in which HCV infection occurs. Altogether, our data provide new insight into the widely used HCV infection model and open a novel perspective for future studies of host-HCV interactions.

## Results

### Identification of small RNA accumulating in HCV-infected and non-infected Huh-7.5 cells

**Fig. 1:**
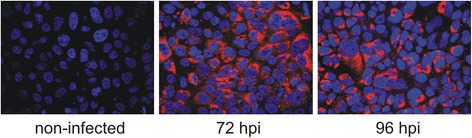
HCV infection in cell cultures. Huh-7.5 cells were inoculated with JFH-1 viral stock at an moi of 1 or 0.1 and cultured for 72 h or 96 h, respectively, when approximately 80% of cells were infected. Non-infected time-matched control cells were cultured in parallel. Cells were visualized via immunofluorescence analysis with mouse monoclonal anti-HCV core antibodies (red) and counterstaining with DAPI to show the locations of nuclei (blue)

To identify and characterize RFs that form during HCV infection we used the HCVcc model. We inoculated Huh-7.5 cells with HCV JFH-1 and collected samples from cultures grown for either 72 h or 96 h post inoculation (hpi). Under the conditions we used, at least 80% of cells were infected at the time of harvest, as shown by immunofluorescence analysis (Fig. 1). Non-infected control cells were cultured and collected in parallel. RNA was isolated from infected (72I, 96I samples) and control (72C, 96C samples) cells. In order to ensure that RFs were generated in cells, and not during the process of sample preparation, stringent criteria of RNA quality control were applied (see Methods). Integrity and purity of the RNA samples were assessed after total RNA isolation and after separation of long and short RNA fractions (the representative electropherograms and RNA integrity numbers (RINs) obtained for the samples subjected to sequencing are shown in Additional file 1: Figure S1). Further analyses involved only those RNA samples for which the RINs obtained for both total and long RNA exceeded 9. In addition, we applied two-dimensional polyacrylamide gel electrophoresis (2D–PAGE) to visualize the global profile of short RNAs accumulation. Previously we showed that this technique allows to effectively monitor changes that various endogenous and exogenous factors induced in the pools of short, high-copy number RNAs (15–80 nt in length) in different types of plant and human cells [6, 15]. We found that in case of the analyzed Huh-7.5 cells, the patterns of short RNA accumulation were reproducible and highly similar across samples (Additional file 1: Figure S2), which again ruled out random RNA degradation during sample handling. Following quality control, the isolated RNA was subjected to next generation sequencing (NGS). Our analysis of RFs was focused on molecules shorter than tRNAs. The size-fractionation applied at the RNA isolation and sequencing library preparation steps was designed in such a way that the surveyed RNA fraction should not contain full-length molecules except for miRNA, siRNA and piRNA. Consequently, other RNAs detected within this length range were assumed to be the longer RNAs’ fragments already present in the starting RNA pool. To identify individual RNA species (RNA molecules with a unique sequence) that ranged from 15 to approximately 80 nucleotides, we performed NGS for 100 sequencing cycles and then subjected the generated reads to rigorous quality filtering. As a result, we were able to determine the entire sequences of 15- to 82-nt-long RNAs without the need to assemble them from shorter reads. In the next step, all identified RNA species were mapped to miRbase, fRNAdb, the HCV JFH-1 genome and the human genome (hg19). The results obtained were manually adjusted, and multiple mappings were resolved via BLAST searches in several databases, including the NCBI, tRNAdb [55] and snoRNA database [56]. The mapping procedure was successful for 5130 cellular RNA species (97%). Next, the levels of their accumulation in each sample were determined (edgeR).

In accordance with our expectations, in addition to miRNA and a small number of full-length mature snoRNA and tRNA, the whole set of the identified molecules included RFs apparently derived from all RNA classes (Additional file 1: Figure S3A). At this stage, full-length snoRNA and tRNA were excluded (147 of 5130 identified species), whereas other small RNAs were divided into 8 groups based on their origin. The highest proportion of RNA species were derived from rRNA (52% of all RNA species; Additional file 1: Figure S3A). However, most of these RNAs accumulated to very low levels. Consequently, the combined amount of all rRNA-derived species did not exceed 15.5% and 8.3% of the normalized read count in the control samples (72C and 96C, respectively, Additional file 1: Figure S3B). This observation clearly indicates that the identified RNAs were not generated by random decay during the sample preparation. If they had been, the combined amount of the rRNA-derived fragments would likely be higher. Importantly, the levels of rRNA fragment accumulation markedly increased upon viral infection, reaching 23.2% and 38.2% in the 72I and 96I samples, respectively (Additional file 1: Figure S3B). The most abundant group in 72C, 72I and 96C samples was miRNA, which accounted for approximately 47%–62% of the normalized read count. In the case of the 96I sample, miRNA was the second most abundant group, after the group of rRNA fragments. The abundance of species derived from tRNA and snoRNA reached 8.6%–12.8% and 6.9%–11.2%, respectively, across samples. The fluctuations in their accumulation levels were not related to the infection. RNA species that represented other groups accumulated to lower levels (Additional file 1: Figure S3B).

### Length distribution of RNA species

**Fig. 2:**
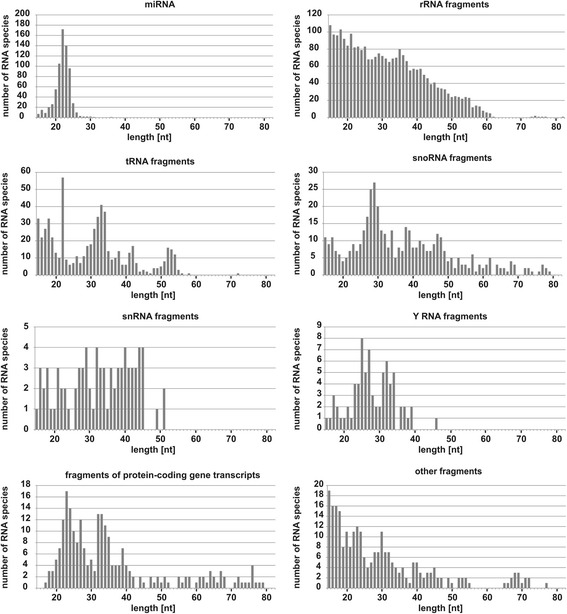
Length distribution plots of small RNA species representing miRNA and the identified groups of RNA fragments

Next, the length distribution of RNA species in all RNA groups was established (Fig. 2). As expected, the majority of miRNAs were between 20 and 24 nt long. The group of fragments derived from rRNA included 2590 species that were from 15 to 82 nt long. We found that the number of species was inversely proportional to their length; the shortest molecules were the most numerous. The group of fragments derived from tRNA included 616 species of 15 to 72 nt in length. Their length distribution plot shows a prominent peak at 22 nt and another two at 31–34 nt and 15–19 nt (Fig. 2). The group of snoRNA-derived fragments included 439 species, from 15 to 79 nt long. For these fragments, three length peaks were also observed: a major one at 26–31 nt and two others at 34 nt and 38–39 nt. The length distribution pattern for snRNA-derived fragments (80 species) did not reveal any prominent peaks – the number of RNA species was similar throughout the length range (15–51 nt), but most of them were between 27 and 45 nt long. Fragments derived from Y RNA (75 species) had a narrower length range, from 15 to 46 nt, with two peaks, one at 23–27 nt and another at 31–34 nt. The RFs that mapped to protein-coding genes or that were classified as “other” (234 and 255 molecules, respectively) also showed specific length distributions.

Altogether, our results indicated that there are some specific rules according to which most RNA classes are cleaved into stable fragments. Only the results obtained for rRNA fragments were clearly different. We assumed that these fragments most likely represented non-specific products of an ongoing digestion process, which is why this group was excluded from further analyses. The distinct size distribution pattern observed for rRNA fragments proves that the specific length peaks detected for other RF species are not related to any technical biases introduced at the library preparation or sequencing stage.

### Characterization of RNA fragments

To identify the basic rules that govern the process of RF formation, we determined the positions where the parental molecules were cut and attempted to establish if the cleavage sites localized to single-stranded or double-stranded regions of the predicted secondary structures of the full-length parental RNAs. In addition, we examined whether RFs comprised elements that would potentially make them competitors of their antecedents. Our analysis was focused on the fragments derived from the following well-defined constitutively expressed non-coding RNA classes: tRNA, snoRNA, snRNA and Y RNA.

**Fig. 3:**
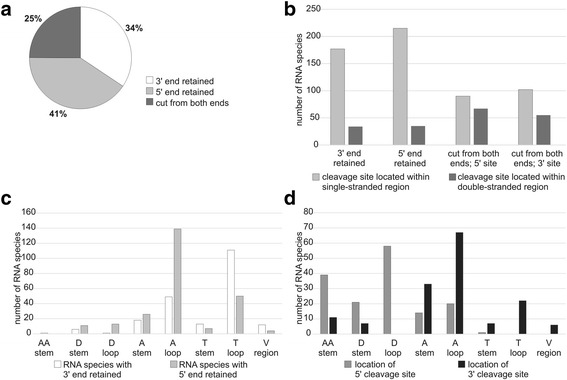
Characterization of tRNA fragments. **a** Proportion of species with a retained 5′ or 3′ end of the parental RNA or those cut from both ends. **b** Number of RNA species generated by cleavage within a single-stranded or double-stranded region of the parental RNA. **c** Distribution of cleavage regions within particular structural elements of mature full-length tRNA cut from one end. **d** Distribution of cleavage regions within particular structural elements of mature full-length tRNA cut from both ends. AA – acceptor arm, D – D arm, A – anticodon arm, T – T arm, V region – variable region. See Additional file 1: Figure S4 for a schematic representation of the mature tRNA structure

**Fig. 4:**
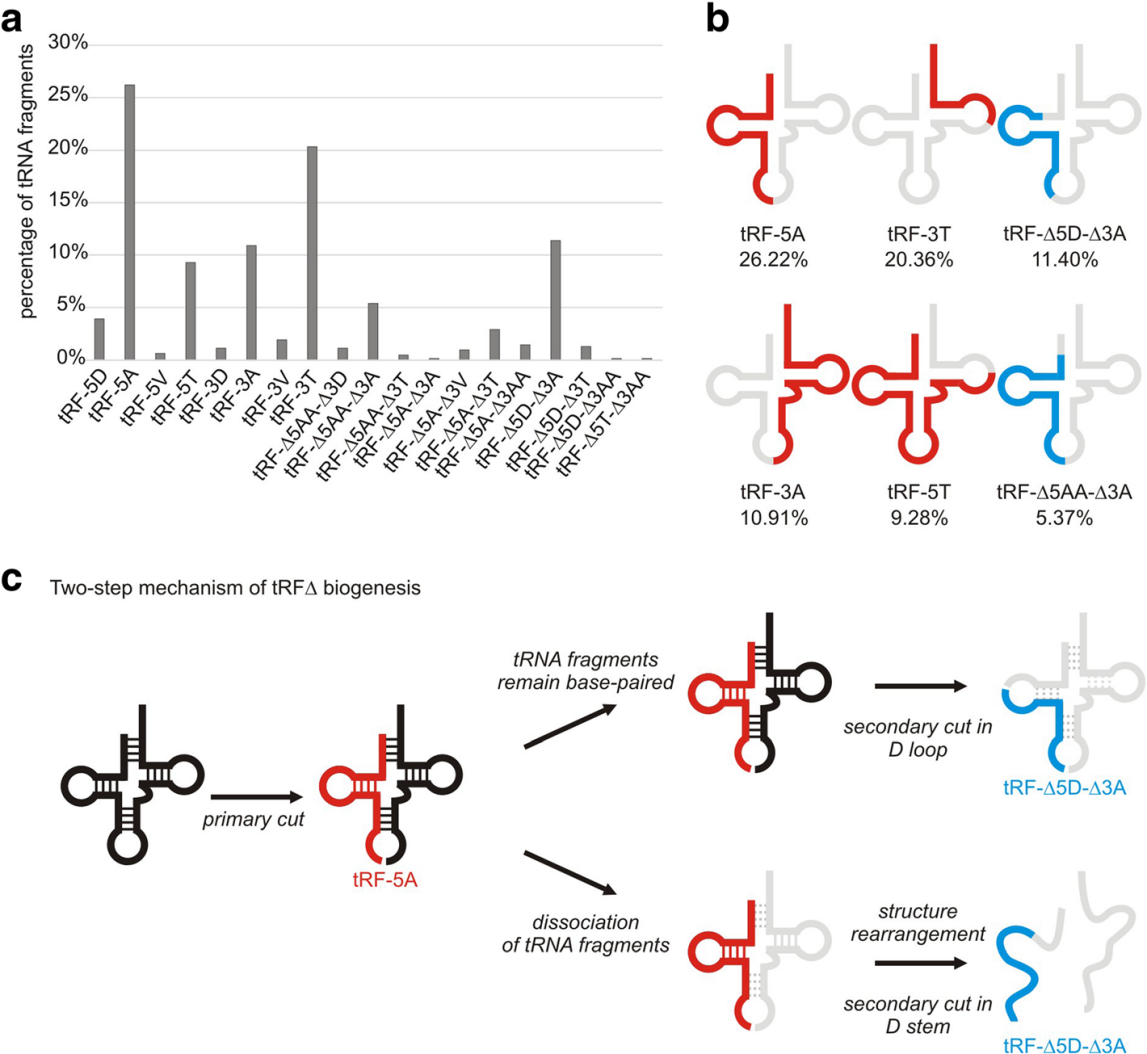
Classes of the identified tRNA fragments (tRFs) and proposed mechanism of their biogenesis. **a** Contribution of particular tRF classes to the entire pool of tRNA derivatives. **b** Schematic representation of the tRFs with frequencies that exceeded 5% of all tRNA derivatives. tRFs are depicted in red (for fragments containing either the 5’ or 3’ end of the parental RNA) or in blue (for fragments cut from both ends), and the lost fragments of parental RNA are in gray. **c** Two-step mechanism of tRFΔ biogenesis. Following a primary cut in a single-stranded region, the resultant fragments can either remain base-paired or dissociate. If the first occurs, the overall fold of the RNA is retained, and a secondary cut takes place within a single-stranded region. In the case of dissociation, the fragments undergo structural rearrangement, which can render the original stems single stranded and thus accessible for a secondary cut. tRFs are depicted in red (for fragments containing either the 5’ or 3’ end of the parental RNA) or in blue (for fragments cut from both ends), and lost fragments of parental RNA are in gray

The majority of tRNA fragments (tRFs) retained either the 5′ or 3′ ends of the mature tRNA (Fig. 3a). They were largely generated by a cleavage within a single-stranded region (Fig. 3b). For tRFs with a retained 5′ end, the cleavage sites were most frequently located in anticodon loop and T loop (Additional file 1: Figure S4, Fig. 3c). A similar cleavage pattern was observed for tRFs that retained the original 3’ end. In this case, however, the majority of cleavage sites were located in the T loop. Notably, 25% of the identified tRFs lacked both ends of the parental molecule (Fig. 3a). In addition, for many of these tRFs, cleavage sites localized to the stems of the full-length tRNA (Fig. 3b). This tendency was especially evident for the 5’ cleavage site, which appeared to be located within a double-stranded region almost as often as in a single-stranded one. Accordingly, the principal 5’ cleavage site of these tRFs was the D loop, followed by the acceptor stem and D stem. 3’ cleavages occurred predominantly in the anticodon loop, anticodon stem and T loop (Fig. 3d). Considering the location of cleavage sites, we divided all tRFs into 19 classes and determined the percentage of RNA species that belong to each particular class. As shown in Fig. 4, the individual classes were named according to the recently proposed general nomenclature of tRFs, where the number refers to the retained end of the mature tRNA (5′ or 3′) and the letter indicates the cleavage site [57]. For example, “tRF-5A” denotes fragments that possess the original 5′ end and were cut in the anticodon arm. Because this nomenclature did not anticipate the formation of molecules cut from both ends, we expanded it with an additional symbol Δ (delta) to indicate that not the entire 5′ or 3′ portion of mature tRNA is retained. For example, “tRF-Δ5D-Δ3A” denotes fragments with a 5′ cleavage site within the D arm and a 3′ cleavage site within the anticodon arm. Among the 19 identified classes of tRFs, 11 included species cut from both ends of mature tRNA, called tRFΔ (Fig. 4a). The dominant class was tRF-5A, which comprised over 26% of all tRNA derivatives. Other fragments with frequencies that exceeded 5% of all tRFs were tRF-3T, tRF-Δ5D-Δ3A, tRF-3A, tRF-5T and tRF-Δ5AA-Δ3A (Fig. 4a,b). Notably, tRF-3T had a highly conservative length of 22 nt and accounted for the previously observed peak in the tRF length distribution plot (Fig. 2). Considering the fact that single-stranded regions are more susceptible to cleavage than double-stranded ones, we hypothesized that tRFΔs are generated according to two alternative mechanisms (Fig. 4c). In both mechanisms, a primary cut occurs within an unpaired region of mature tRNA, predominantly in the anticodon loop. The first mechanism assumes that the two resultant fragments of tRNA remain base-paired, which preserves the overall folding pattern of the molecule. In such a situation, a secondary cut takes place within another single-stranded region, primarily in the D loop. The second mechanism assumes that both parts of the nicked tRNA dissociate and subsequently undergo structural rearrangements. Consequently, the originally helical regions can become single-stranded, which are more accessible for a secondary cut. This mechanism well explains the significant increase in cleavage frequency within the initially double-stranded regions observed for tRFΔs.

**Fig. 5:**
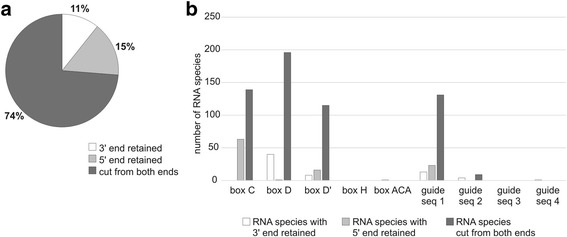
Characterization of snoRNA fragments (snoRFs). **a** Proportion of species with a retained 5’ or 3’ end of the parental RNA or those cut from both ends. **b** Functionally relevant regions of mature snoRNA included in the snoRFs. See Additional file 1: Figure S5 for a simplified representation of the mature snoRNA structure

In contrast to tRFs, most of snoRNA fragments (snoRFs) (74%) lacked the original 5′ and 3′ ends of their parental molecules (Fig. 5a). Because of the structural diversity of snoRNA, we did not analyze the location of cleavage sites within secondary structures. Instead, we examined whether snoRFs contained functionally relevant regions of snoRNA (Additional file 1: Figure S5). The majority of snoRFs (409) were derived from C/D box snoRNAs. Nearly all of them carried at least one of the boxes (C, D, and/or D′) and/or a guide sequence (Fig. 5b). Among the 11 snoRFs originating from H/ACA box snoRNA, 1 included an ACA box and 4 included a guide sequence. Similarly, 9 of 18 snoRFs derived from small Cajal body-specific RNAs contained a guide sequence, which in some cases was also accompanied by box C and/or D’. In general, most snoRFs, cut either from one or both ends, retained box D (238 individual species). Box C, box D’ and a guide sequences were present in 203, 140 and 182 snoRFs, respectively.

**Fig. 6:**
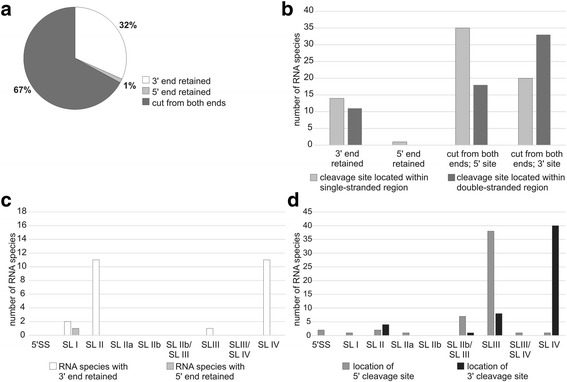
Characterization of snRNA fragments (snRFs). **a** Proportion of species with a retained 5’ or 3’ end of the parental RNA or those cut from both ends. **b** Number of species generated by cleavage within a single-stranded or double-stranded region of the parental RNA. **c** Distribution of cleavage regions within particular structural elements of mature full-length snRNA cut from one end. **d** Distribution of cleavage regions within particular structural elements of mature full-length snRNA cut from both ends. 5’ SS – 5’ single-stranded region, SL – stem-loop (I through IV), SLIIb/SLIII – single-stranded region between stem-loop II and stem-loop III, SLIII/SLIV – single-stranded region between stem-loop III and stem-loop IV. See Additional file 1: Figure S6 for a schematic representation of the mature snRNA structure

The majority of snRNA fragments (snRFs) (67%) were generated by a cleavage from both ends of their parental molecules (Fig. 6a), whereas 32% of snRFs retained the original 3’ end, and only 1% had the 5’end of mature snRNA. In general, the cleavages appeared to occur in single-stranded and double-stranded regions with comparable frequencies. A more detailed analysis confirmed that this was true for snRFs with the 3′ end retained (Fig. 6b). However, in the case of snRFs generated by cutting both ends of the mature snoRNA, the 5’ cleavage site was predominantly located within unpaired regions. In contrast, 3′ cleavage sites were more often localized to the helices. This observation may indicate that the biogenesis of this subset of snRFs proceeds according to the model proposed for tRFΔs and involves primary and secondary cuts. The primary 5′ cleavage occurs in single-stranded regions. Next, the resultant RF undergoes a structural rearrangement that exposes the 3′ cleavage site, originally located within a base-paired region of a parental full-length snRNA. Most snRFs were derived from U1 snRNA (47 species), followed by those from U2 snRNA (18), U4 snRNA (10), U5 snRNA (4), and U4atac snRNA (1). Stem-loops II and IV (SL II and SL IV) (Additional file 1: Figure S6) were the most frequent cleavage sites for fragments with a retained 3′ end (Fig. 6c). The major 5′ and 3′ cleavage sites for snRFs cut from both ends of the mature snRNA were SL III and SL IV, respectively (Fig. 6d). Mature snRNAs comprise several segments of special functional significance; for example those involved in hybridization with 5′ splice sites or branch points (Additional file 1: Figure S6). Our analysis revealed that among such segments, only those engaged in interactions with Sm proteins were present in snRFs (in 56 of 80 species).

**Fig. 7:**
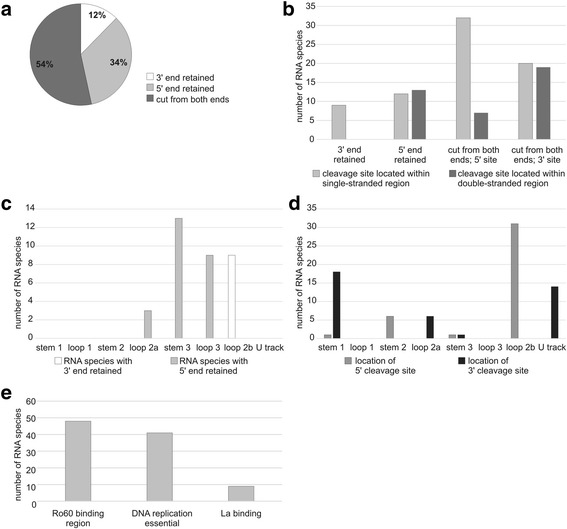
Characterization of Y RNA fragments (YRFs). **a** Proportion of species with a retained 5′ or 3′ end of the parental RNA or those cut from both ends. **b** Number of species generated by cleavage within a single-stranded or double-stranded region of the parental RNA. **c** Distribution of cleavage regions within particular structural elements of mature full-length Y RNA cut from one end. **d** Distribution of cleavage regions within particular structural elements of mature full-length Y RNA cut from both ends. See Additional file 1: Figure S7 for a schematic representation of mature Y RNA structure. **e** Functionally relevant regions of mature Y RNA included in YRFs

Approximately one-third of Y RNA fragments (YRFs) retained the 5’ end of mature Y RNA (Fig. 7a). They were generated by cleavage within stem 3, loop 3 and loop 2a (Additional file 1: Figure S7), with no trend for the cleavage site being located within single-stranded or double-stranded regions (Fig. 7bc,). 12% of YRFs had the original 3’ end. All of them were cut in loop 2b, which indicates a remarkably specific biogenesis. Fragments cut from both ends of mature Y RNA had a major 5’ cleavage site within loop 2b and two main 3′ cleavage sites, within stem 1 and the U track (Fig. 7d). Whereas 5’ cleavage sites predominantly localized to a single-stranded region, 3′ cuts occurred with similar frequencies in single-stranded and base-paired regions. Again, this observation suggests a biogenesis in accord with the model proposed for tRFΔs. Most YRFs (37 species) originated from hY4. In addition, hY1, hY5 and hY3 gave rise to 18, 11 and 7 RNA species, respectively. Mature Y RNAs perform their functions via binding with Ro60 and La proteins. In addition, they also have regions essential for DNA replication [58]. The majority of YRFs (48 species) comprised segments involved in interactions with Ro60. Regions implicated in DNA replication were present in 41 YRFs, and 9 species had La binding site (Fig. 7e).

### Identification of RNA cleavage patterns and selection of representative RFs

To determine the patterns of the cleavage of parental RNA into fragments, we aligned the sequences of all identified RNA species to the sequences of their parental molecules. As a result, we identified three major cleavage patterns.

**Fig. 8:**
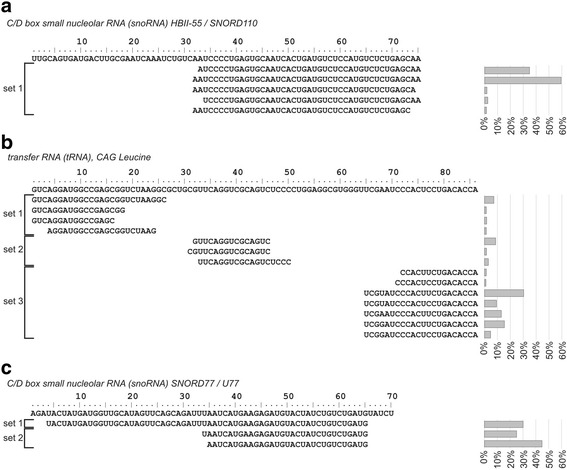
Three major patterns of parental RNA cleavage: single-set single-region (**a**), single-set many-regions (**b**), many-sets single-region (**c**). Sequences of RFs are aligned to the sequence of their parental RNA. The frequencies of particular RFs derived from a single full-length RNA are presented on the right, while the classification to the sets is depicted on the left

The first pattern, called a single-set single-region pattern, was observed when all RFs were of similar length and overlapped a single region of parental RNA (Fig. 8a). There were significant differences in the frequencies of particular RFs in the set – several of them, usually two, were highly pronounced, whereas the others were rare. This pattern is similar to the way in which some miRNAs are cut out from their precursors. As a result, the phenomenon of end heterogeneity is observed [59].

The second pattern, called a single-set many-regions pattern, was observed when RFs were excised from more than one region of parental RNA and constituted separate sets of RFs, each overlapping another portion of the full-length molecule (Fig. 8b). Each set had a considerably more frequent master RF, accompanied by a spectrum of less pronounced RFs.

The third pattern, called a many-sets single-region pattern, was observed when one region of parental RNA gave rise to at least two sets of RFs of substantially different lengths (Fig. 8c). In some cases (as depicted in Fig. 8c), shorter fragments were approximately twice as frequent as the longer ones, which suggests that the latter may have been cut to yield the former.

A detailed examination of the composition of the sets revealed that they comprised one or more clusters of very similar species. For example, the species differed only by a few nucleotides in length or had single nucleotide substitutions. The latter were detected mainly in the case of tRFs and could be attributed to the base modifications of mature tRNA. It is known that RNA modifications can decrease the fidelity of cDNA synthesis during the preparation of sequencing libraries [60]. We assumed that the highly similar species may additively constitute a nearly homogeneous population of molecules that may not be distinguishable by the cellular machinery. Therefore, we decided that for further analysis, it would be useful to select one species as a representative of each cluster. This approach should allow for a transition from a dataset in which the focus is on all possible RNA species to one in which biological significance is the central point. Representative RFs and miRNA (for clarity denoted in italics – *RFs, miRNA*) were selected as described in Methods.

### *RFs* are as abundant as *miRNA* in Huh-7.5 cells

**Fig. 9:**
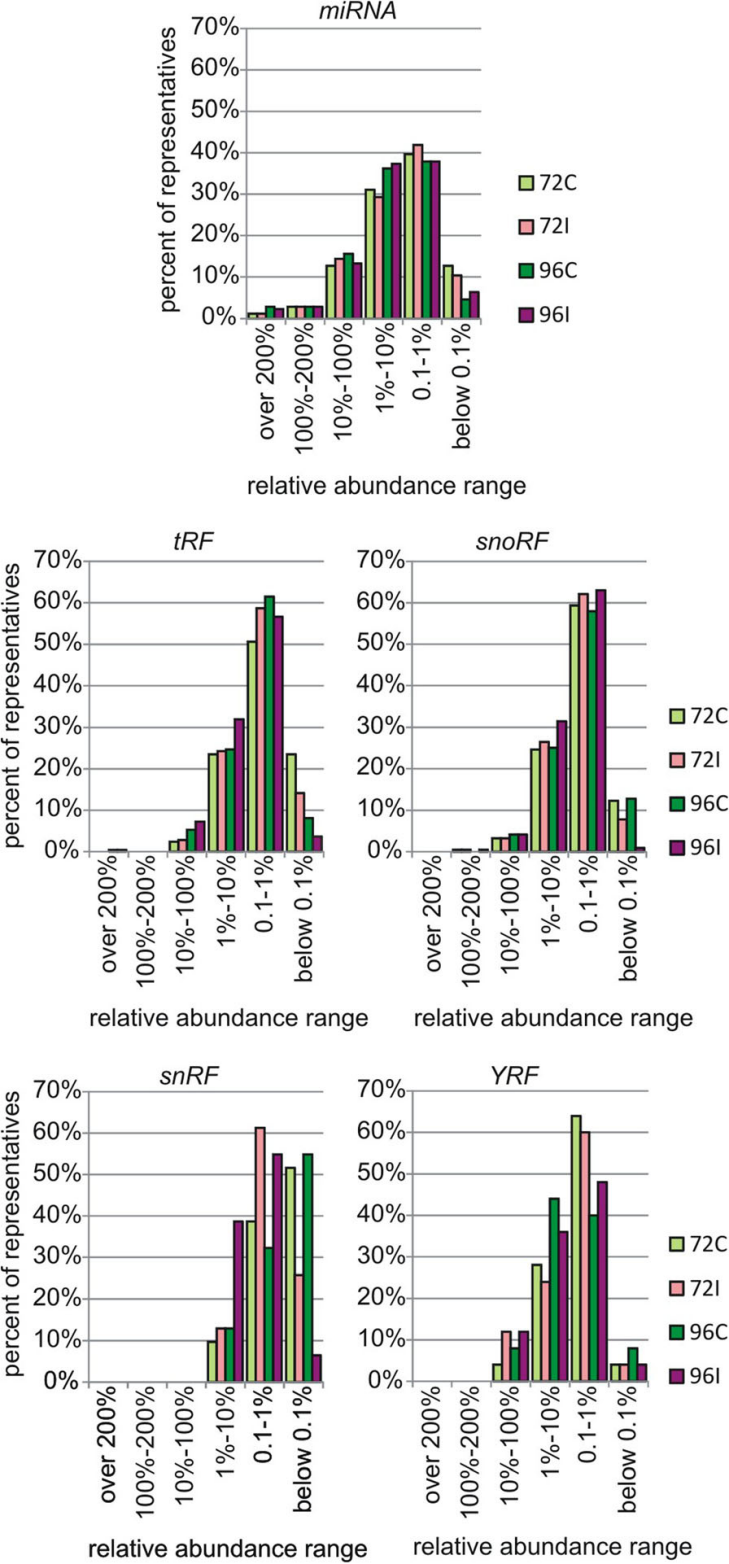
Relative abundance of representative miRNA and RNA fragments. The abundance of *miRNA* and *RFs* in all samples (72C, 72I, 96C and 96I) was established as a value relative to the amount of liver-specific *miR-122* (*miR-122* content was considered as 100%). All analyzed RNAs were assigned to appropriate relative abundance ranges based on their accumulation levels

Next, the abundance of *miRNA* and *RFs* in all samples was established as a value relative to the amount of liver-specific *miR-122* (the mean level of accumulation of the latter in 72C, 72I, 96C and 96I samples was considered 100%) (Fig. 9). Six ranges of relative abundance were designated: over 200%, 100%–200%, 10%–100%, 1%–10%, 0.1%–1% and below 0.1%. All analyzed RNAs were assigned to appropriate ranges based on their accumulation levels. For approximately 40% of the *miRNAs,* their relative abundances were in the range 0.1%–1%, which meant that they accumulated to levels two or three orders of magnitude lower than *miR-122*. For similar numbers of *miRNA,* their relative abundances were in the range 1%–10%. Only approximately 5% of *miRNAs* reached or exceeded the accumulation level of *miR-122* (ranges 100%–200% and above 200%).

More than a half of *tRFs* was assigned to the range 0.1%–1%. Further, 23%–32% of *tRFs* displayed relative abundance of 1%–10%, whereas for 2%–7%, their relative abundances were in the range 10%–100%. Several *tRFs* accumulated to higher levels than *miR-122*. The relative abundance of *tRFs* in cultured cells increased over time and was higher upon HCV infection. Similar results were obtained for *snoRFs* and *YRFs*. A lower relative abundance was observed for *snRFs*. In non-infected cells, over half of *snRFs* were assigned to the range below 0.1%, and the other half was split between the 0.1%–1% and 1%–10% ranges. However, HCV infection triggered a substantial increase in snRNA fragmentation, which was most evident at 96 hpi. Taken together, the distribution of the relative abundance values in the analyzed classes of *RFs* greatly resembled the one obtained for *miRNA*. This observation indicates that RNA fragments are as abundant as miRNA in the HCV cell culture model.

All of the 25 most abundant *RFs* were assigned to a relative abundance range of at least 1%–10% across the samples, and the majority were assigned to the 10%–100% range (Table 1). Among them, 10 originated from tRNA and included: 7 *tRF-5A*, 2 *tRF-5T* and 1 *tRF-Δ5T-Δ3AA*. tRNA-Val and tRNA-Gly were the major parental molecules of *tRF-5A*. tRNA-Gly also gave rise to both *tRF-5T*, whereas tRNA-Thr yielded *tRF-Δ5T-Δ3AA*. Furthermore, 7 highly accumulated *RFs* were derived from C/D box snoRNA: SNORD30, SNORD44, SNORD58, HBII-420/SNORD99, SNORD78 and SNORD81. All of them included at least one of the functionally relevant segments (i.e., box C, D, D′ or a guide sequence). Y RNA generated 2 plentiful fragments, both containing an Ro60 binding region. We also identified 1 abundant fragment of HIST2H2AA3 mRNA, 1 tRNAseZ^L^-interacting RNA and 5 fragments classified as derivatives of putative conserved non-coding RNA. The length of the highly accumulated *RFs* spanned a range from 15 to 69 nt. Two of them, derived from Y RNA and putative conserved non-coding RNA, had sizes similar to those of mi/siRNA (23 and 24 nt, respectively).

**Table 1:** Features of the 25 most abundant representative RNA fragments

Sequence	Length	Parental RNA	RNA class	Fragment type	Relative abundance			
					72C	72I	96C	96I
CACCACGUUCCCGUGG	16	Putative conserved non-coding region (RNAz)	other	n/a^b^	164.31%	137.42%	97.74%	81.31%
GUUUGUGAUGACUUACA	17	C/D box snoRNA, SNORD30/U30	snoRNA	box C	147.21%	109.31%	73.54%	139.20%
UCGUACGACUCUUAGCGG	18	*Homo sapiens* tRNaseZ^L^-interacting RNA B2	other	n/a	106.20%	77.68%	59.03%	112.86%
GUUUCCGUAGUGUAGUGGUUAUCACGUUCGCCU	33	tRNA, AAC (Val)	tRNA	tRF-5A	94.61%	85.11%	226.16%	296.63%
CUGGAUGAUGAUAAGCAAAUGCUGACUGAAC	31	C/D box snoRNA, SNORD44/U44	snoRNA	box C	49.19%	35.07%	36.15%	41.96%
ACCACGUUCCCGUGG	15	Putative conserved non-coding region (RNAz)	other	n/a	39.10%	39.74%	47.31%	33.02%
GUUUCCGUAGUGUAGUGGUUAUCACGUUCGCCUC	34	tRNA, CAC (Val)	tRNA	tRF-5A	30.75%	15.72%	51.93%	31.72%
UUGCUGUGAUGACUAUCUUAGGACACCUU	29	C/D box snoRNA, SNORD58/U58	snoRNA	box C, guide sequence 1	28.55%	20.87%	13.10%	22.32%
CCUGUGAACUCAAAAGGCUCUUUUCAGAGCCACU	34	mRNA, HIST2H2AA3	mRNA	n/a	26.32%	17.69%	13.46%	12.49%
GGUCCAGGAUGAAACCUAAUUUGAGUGGACAUCCAUGGAUGAGAAAUGCGGAUAUGGGACUGAGA	65	C/D box snoRNA, HBII-420/SNORD99	snoRNA	box C, D, guide sequence 1	26.20%	24.58%	24.72%	27.16%
GUACGACUCUUAGCGG	16	tRNA, AGT (Thr)	tRNA	tRF-Δ5T-Δ3AA	24.37%	19.51%	17.18%	32.54%
CCUGGAUGAUGAUAAGCAAAUGCUGACU	28	C/D box snoRNA, SNORD44/U44	snoRNA	box C	18.02%	16.31%	27.18%	28.47%
GCCCGGCUAGCUCAGUCGGUAGAGCAUGAGACUC	34	tRNA, CTT (Lys)	tRNA	tRF-5A	17.80%	8.89%	24.82%	15.98%
UGAGCAUGUAGACAAAGGUAACACUGAAG	29	C/D box snoRNA, SNORD78/U78	snoRNA	box D	17.68%	18.08%	20.05%	24.69%
GCAUUGGUGGUUCAGUGGUAGAAUUCUCGCCUCCCACGCGGGAGACCCGGGU	52	tRNA, CCC (Gly)	tRNA	tRF-5T	15.24%	6.89%	11.55%	15.44%
AAUACAUGAUGAUCUCAAUCCAACUUGAACUCUCUCACUGAUUACUUGAUGACAAUAAAAUAUCUGAUA	69	C/D box snoRNA, SNORD81/U81/Z23	snoRNA	box C, D, D’, guide sequence 1	14.77%	18.35%	12.49%	16.56%
UAUUGCACUUGUCCCGGCCUGUUA	24	Putative conserved non-coding region (RNAz)	other	n/a	13.01%	13.20%	15.07%	13.80%
GGCUGGUCCGAUGGUAGUGGGUUAUCAGAACU	32	hy4 Ro RNA	Y RNA	Ro60 binding region	11.65%	29.69%	15.19%	38.64%
GCAUUGGUCGUUCAGUGGUAGAAUUCUCGCCU	32	tRNA, CCC (Gly)	tRNA	tRF-5A	11.58%	16.71%	31.53%	58.67%
UCCACCACGUUCCCGUGG	18	Putative conserved non-coding region (RNAz)	other	n/a	9.42%	12.81%	18.59%	8.16%
GCAUUGGUGGUUCAGUGGUAGAAUUCUCGCCUGC	34	tRNA, GCC (Gly)	tRNA	tRF-5A	9.37%	6.50%	21.50%	20.20%
GCUUCUGUAGUGUAGUGGUUAUCACGUUCGCCU	33	tRNA, CAC (Val)	tRNA	tRF5A	9.04%	6.33%	19.65%	21.11%
GCAUUGUGGUUCAGUGGUAGAAUUCUCGC	29	tRNA, GCC (Gly)	tRNA	tRF-5A	6.58%	10.57%	15.96%	36.65%
GCAUUGGUGGUUCAGUGGUAGAAUUCUCGCCUGCCACGCGGGAGGCCCGGGU	52	tRNA, GCC (Gly)	tRNA	tRF-5T	4.78%	5.57%	10.11%	21.10%
CCCCCCACUGCUAAAUUUGACUG^a^	23	hy4 Ro RNA	Y RNA	Ro60 binding region	4.03%	16.11%	5.51%	44.43%

^a^differentially accumulated at 96 hpi

^b^not applicable

### HCV infection increases the accumulation of low copy number RNA fragments

To investigate whether HCV infection induces changes in the pool of *RFs*, we compared *RF* accumulation levels in infected and non-infected Huh-7.5 cells. The analysis revealed that the accumulation of a vast majority of *RFs* was not affected. The molecules that did display statistically significant differential accumulation between infected and control cells were the *RFs* of low abundance. Among 827 representative species, 62 and 96 were found to be differentially accumulated at 72 hpi and 96 hpi, respectively, with 52 in common for both time points. Table 2 presents the 25 most abundant differentially accumulated *RFs*. The relative abundances of all of them were in the ranges 0.1%–1% and 1%–10% in infected cells at 72 hpi and 96 hpi, respectively. The most prominent differentially accumulated *RF* originated from Y RNA. It demonstrated an increased abundance upon HCV infection at 72 hpi and 96 hpi, but this change reached statistical significance only at the latter time point (log_2_FC ≥ 2, FDR < 0.05). This Y RNA-derived *RF*, mentioned above as one of the most abundant fragments (Table 1), contained an Ro60 binding region and displayed miRNA-like length (23 nt). The 25 most plentiful fragments with differential accumulation (Table 2) also included *RFs* derived from tRNA (9), snoRNA (5), snRNA (5), piRNA (1) and 3 molecules that could not be unambiguously classified. The predominant *tRFs* were *tRF-5A*, which originated from tRNA-Glu, tRNA-Leu and tRNA-Tyr. tRNA-Glu and tRNA-Leu were also the source of *tRF-5D*, and tRNA-Glu was the source of *tRF-Δ5A-Δ3AA*. The remaining 2 most abundant differentially accumulated *tRFs* were *tRF-Δ5D-Δ3A* and *tRF->5T*, derived from tRNA-Gln and tRNA-Lys, respectively. The snoRNAs whose fragmentation was significantly elevated upon HCV infection included SNORD82, SNORD26, SNORD45, SNORA7 and small Cajal body-associated HBII-382. Nearly all of them yielded fragments that contained functionally relevant regions such as box C, D, and ACA and/or a guide sequence. HCV infection was also associated with an increased production of fragments originating from snRNA, in particular from U1, which gave rise to 4 *snRFs* of considerable abundance, 3 of which contained an Sm binding site. One U2-derived *RF* was also among the 25 most abundant differentially accumulated *RFs*. The lengths of all *RFs* classified in this group ranged between 15 and 55 nt, with 4 representative species (derived from Y RNA and snoRNA) displaying miRNA-like lengths of 21 to 24 nt.

**Table 2:** Features of the 25 most abundant differentially accumulated representative RNA fragments

Sequence	Length	Parental RNA	RNA class	Fragment type	log_2_FC 72 hpi^a^	log_2_FC 96 hpi^a^	Relative abundance			
							72C	72I	96C	96I
CCCCCCACUGCUAAAUUUGACUG	23	hy4 Ro RNA	Y RNA	Ro60 binding region	2.00	**3.01**	4.03%	16.11%	5.51%	44.43%
UCCCACAUGGUCUAGCGGUUAGGAUUCCUGGUU	33	tRNA, TTC (Glu)	tRNA	tRF-5A	2.14	**2.43**	2.10%	9.25%	2.76%	14.90%
UCCCUGGUGGUCUAG	15	tRNA, CTC (Glu)	tRNA	tRF-5D	**3.04**	1.41	0.27%	2.25%	0.86%	2.29%
UACGACUCUUAGCGGUGGAUCACUCGGC	28	*ambiguous mapping (intergenic / rRNA)*	other	n/a^b^	**2.38**	1.95	0.20%	1.03%	0.30%	1.16%
GCCCGGCUAGCUCAGUCGGUAGAGCAUGGGACUCUUAAUCCCAGGGUCGUGGGUU	55	tRNA, CTT (Lys)	tRNA	tRF-5T	1.03	**2.40**	0.48%	0.98%	0.44%	2.34%
GUUAAGAUGGCAGAGCCCGGUAAUCGCAUAAAACUUAAAACU	42	tRNA, TAA (Leu)	tRNA	tRF-5A	0.93	**2.54**	0.50%	0.96%	0.54%	3.13%
UUGGUCGUGGUUGUAGUCCGUGCGAGAA	28	tRNA, TTC (Glu)	tRNA	tRF-Δ5A-Δ3AA	1.36	**4.32**	0.34%	0.86%	0.26%	5.24%
GUUCGCGCUUUCCCCUG	17	U1 spliceosomal RNA	snRNA	no functional region	0.61	**3.56**	0.54%	0.83%	0.21%	2.46%
CGGCCACUGAUUAUCGAGGCGAUUCUGAUCUG	32	scaRNA, HBII-382/scaRNA2	scaRNA	no functional region	0.80	**2.62**	0.46%	0.80%	0.57%	3.52%
CCCCACUGCUAAAUUUGACUG	21	hy4 Ro RNA	Y RNA	Ro60 binding region	**2.63**	**3.70**	0.12%	0.72%	0.25%	3.18%
ACUCGACUGCAUAAUUUGUGGUAGUGGGG	29	U1 spliceosomal RNA	snRNA	Sm binding	**5.22**	**7.82**	0.02%	0.58%	0.03%	7.43%
UAGGAUGGGGUGUGAUAGGUGGCACGGAGAA	31	*ambiguous mapping (intergenic / tRNA)*	other	n/a	1.38	**3.55**	0.21%	0.54%	0.17%	2.05%
CACAAAUGAUGAAUAACAAAGGGACU	26	C/D box snoRNA, SNORD82/U82	snoRNA	box C	**2.40**	**3.49**	0.10%	0.53%	0.15%	1.72%
UCCCUGGUGGUCUAGUGGUUAGGAUUCGGCGCUCUCACC	39	tRNA, TTC (Glu)	tRNA	tRF-5A	1.62	**2.60**	0.17%	0.51%	0.22%	1.35%
GGUUAGCACUCUGGACUC	18	tRNA,CTG (Gln)	tRNA	tRF-Δ5D-Δ3A	**3.02**	**2.46**	0.06%	0.49%	0.11%	0.63%
UCGUACGACUCUUAGCGGUGGAUCACUCGGC	31	*ambiguous mapping (intergenic / rRNA)*	other	n/a	1.98	**2.37**	0.12%	0.46%	0.14%	0.74%
AAACUCGACUGCAUAAUUUGUGGUAGUGGGGGACU	35	U1 spliceosomal RNA	snRNA	Sm binding	**5.70**	**9.02**	0.01%	0.40%	0.01%	5.00%
GUUAAGAUGGCAGAGCCC	18	tRNA, TAA (Leu)	tRNA	tRF-5D	2.07	**2.31**	0.09%	0.38%	0.22%	1.07%
GGUAAAAUGGCUGAGUGAAGCAUUGGACU	29	tRNA, GTA (Tyr)	tRNA	tRF-5A	1.33	**2.65**	0.14%	0.36%	0.17%	1.05%
ACUUUAGCUCUAGAAUUACUCUGAGACCU	29	C/D box snoRNA, SNORD45/U45	snoRNA	box D, guide sequence 2	2.00	**5.05**	0.09%	0.35%	0.06%	2.14%
CUACGGGGAUGAUUUUACGAAC	22	C/D box snoRNA, SNORD26/U26	snoRNA	box C	**3.05**	**2.40**	0.04%	0.35%	0.11%	0.58%
UCACCCGGCCCGGACACG	18	piRNA	other	n/a	**5.07**	**8.18**	0.01%	0.34%	0.01%	4.13%
AAUGUGGGAAACUCGACUGCAUAAUUUGUGGUAGUGGGGGACU	43	U1 spliceosomal RNA	snRNA	Sm binding	**8.36**	**9.93**	0.00%	0.34%	0.00%	3.09%
AUUGGAAGACACUCUGCGACAGUG	24	H/ACA box snoRNA, ACA7/SNORA7/SNORA7A	snoRNA	box ACA, guide sequence 4	0.97	**3.22**	0.17%	0.33%	0.18%	1.64%
CACGCAUCGACCUGGUAUUGCAGUACCUCCAGGAACGG	38	U2 spliceosomal RNA	snRNA	no functional region	**2.62**	**5.47**	0.05%	0.31%	0.04%	1.78%

^a^differentially accumulated (log_2_FC ≥ 2 or ≤ −2 and FDR < 0.05; relative abundance sorted by 72I) are marked with boldtype

^b^not applicable

In the next stage of our analysis, we focused on the *RFs* with the highest fold change upon HCV infection. Their relative abundance in non-infected cells was extremely low and did not reach 0.1% of the amount of *miR-122*. However, HCV infection dramatically raised their accumulation levels – in some cases the change was several orders of magnitude. *RFs* with log_2_FC ≥ 5, at least at 96 hpi, are presented in Table 3. Among the 26 such *RFs*, 10 were generated from U1 snRNA and 1 from U2 snRNA. This indicates that spliceosomal RNAs are predominant targets of the RNA cleavage that occurs in cells upon HCV infection. Other *RFs* were derived from snoRNA (6), tRNA (2), putative conserved noncoding regions (2) and piRNA (2). In addition, this group included derivatives of 3 parental molecules that otherwise did not yield any other fragments: fibroblast growth factor-2 (FGF-2) internal ribosome entry site (IRES), signal recognition particle RNA (7S RNA) and nuclear RNase P RNA.

**Table 3:** Features of the 26 representative RNA fragments with log_2_FC > 5^a^

Sequence	Length	Parental RNA	RNA class	Fragment type	log_2_FC 72 hpi^b^	log_2_FC 96 hpi^b^	Relative abundance			
							72C	72I	96C	96I
AAACUCGACUGCAUAAUUUGUGGUAGUGGGGGACUG	36	U1 spliceosomal RNA	snRNA	Sm binding	**5.14**	**11.59**	0.00%	0.01%	0.00%	0.26%
AUGUGGGAAACUCGACUGCAUAAUUUGUGGUAGUGGGGGA	40	U1 spliceosomal RNA	snRNA	Sm binding	**7.27**	**11.33**	0.00%	0.01%	0.00%	0.21%
AAUGUGGGAAACUCGACUGCAUAAUUUGUGGUAGUGGGGGACU	43	U1 spliceosomal RNA	snRNA	Sm binding	**8.36**	**9.93**	0.00%	0.34%	0.00%	3.09%
ACCCCACGUCUCGUCGCG	18	FGF-2 internal ribosome entry site (IRES)	other	n/a^c^	**6.54**	**9.45**	0.00%	0.04%	0.00%	0.26%
GGUCCGCCGGCCCUG	15	Putative conserved non-coding region (EvoFold)	other	n/a	**6.90**	**9.35**	0.00%	0.05%	0.00%	0.27%
AAACUCGACUGCAUAAUUUGUGGUAGUGGGGGACU	35	U1 spliceosomal RNA	snRNA	Sm binding	**5.70**	**9.02**	0.01%	0.40%	0.01%	5.00%
ACUCGACUGCAUAAUUUGUGGUAGUGGGGGACUG	34	U1 spliceosomal RNA	snRNA	Sm binding	**5.67**	**9.00**	0.00%	0.19%	0.01%	2.55%
AUGUGGGAAACUCGACUGCAUAAUUUGUGGUAGUGGGG	38	U1 spliceosomal RNA	snRNA	Sm binding	**6.71**	**8.92**	0.00%	0.25%	0.01%	2.89%
CUGGCAGGGGAGAUACCAUGAUCACGAAGGUGGUUUUCCCAGGGC	45	U1spliceosomalRNA	snRNA	no functional region	**6.49**	**8.45**	0.00%	0.07%	0.00%	0.93%
GAGUUCUGGGCUGUAGUGCGCU	22	7S RNA	other	n/a	**4.67**	**8.33**	0.00%	0.06%	0.00%	0.29%
UGGGCAGGAGAUGCCGUGGACCCC	24	Nuclear RNase P	other	n/a	**5.22**	**8.18**	0.00%	0.03%	0.00%	0.29%
UCACCCGGCCCGGACACG	18	piRNA	other	n/a	**5.07**	**8.18**	0.01%	0.34%	0.01%	4.13%
GGGACUGACCUGAAAUGAAGAGAAUACU	28	C/D box snoRNA, SNORD2/snR39B	snoRNA	box D’	**4.20**	**7.88**	0.00%	0.02%	0.00%	0.30%
ACUCGACUGCAUAAUUUGUGGUAGUGGGG	29	U1 spliceosomal RNA	snRNA	Sm binding	**5.22**	**7.82**	0.02%	0.58%	0.03%	7.43%
AUUGCACUCCGGAUGUGCUGACCCCU	26	U1 spliceosomal RNA	snRNA	no functional region	**4.88**	**6.87**	0.00%	0.06%	0.00%	0.46%
AUUGCACUCCGGAUGUGCUGACCCCUGCGAUUUCCCCAAAUGUGG	45	U1 spliceosomal RNA	snRNA	no functional region	**4.21**	**6.49**	0.00%	0.04%	0.00%	0.26%
UCACCCGGCCCGGAC	15	piRNA	other	n/a	**5.02**	**6.31**	0.01%	0.25%	0.02%	1.76%
ACCCAGGCGGCCCGGGUUCGACUCCCGGUGUG	32	tRNA, TTC (Glu)	tRNA	tRF-Δ5A-Δ3AA	**5.62**	**6.14**	0.00%	0.04%	0.00%	0.30%
GCGCGCCGGCCGGGCG	16	Putative conserved non-coding region (EvoFold)	other	n/a	**4.26**	**5.69**	0.01%	0.26%	0.04%	1.89%
UUUUACGGAUCUGGCUUCUGAGA	23	C/D box snoRNA, SNORD50/U50A	snoRNA	box D, guide sequence	1.36	**5.65**	0.03%	0.07%	0.01%	0.57%
CACGCAUCGACCUGGUAUUGCAGUACCUCCAGGAACGG	38	U2 spliceosomal RNA	snRNA	no functional region	**2.62**	**5.47**	0.05%	0.31%	0.04%	1.78%
UAGCUCUAGAAUUACUCUGAGACCU	25	C/D box snoRNA, SNORD45/U45	snoRNA	box D	0.90	**5.30**	0.03%	0.05%	0.01%	0.31%
AUACAUGAUGAUCUCAAUCCAACUUGAACUCU	32	C/D box snoRNA, SNORD81/U81/Z23	snoRNA	box C	**2.82**	**5.13**	0.01%	0.06%	0.01%	0.34%
AAUCUGUAGUCUUGGAGCCGCACAGGGUUGGUGGUACCCUCG	42	scaRNA, scaRNA13/U93	snoRNA	no functional region	1.72	**5.11**	0.03%	0.11%	0.02%	0.85%
ACUUUAGCUCUAGAAUUACUCUGAGACCU	29	C/D box snoRNA, SNORD45/U45	snoRNA	box D, guide sequence	2.00	**5.05**	0.09%	0.35%	0.06%	2.14%
AGAAAUAUGUCUGAUAAAAGAUUUACUUUGAUAGAGUAAAUAAUAGGAGCU	51	tRNA, GAT (Ile)	tRNA	tRF-5T	1.47	**5.00**	0.02%	0.07%	0.01%	0.33%

^a^at least at 96 hpi (FDR < 0.05; sorted by log_2_FC at 96 hpi)

^b^differentially accumulated are marked with boldtype

^c^not applicable

Collectively, our analyses reveal that HCV infection triggers an overall increase in the accumulation of RFs. The infection, however, mostly impacts the fraction of low copy number fragments. Therefore, even upon up-regulation, most of these differentially accumulated RNAs remained less abundant than the fragments that were reproducibly generated in high amounts, both in infected and non-infected cells.

## Discussion

RFs are an emerging group of non-coding RNAs with functional potential. Although they have been increasingly well characterized in a variety of organisms under both pathological and physiological conditions [5, 15, 27–30, 61], only a few reports have addressed the problem of the formation and significance of RFs in the context of viral infection [31–35]. In this study, we characterized the RFs that are generated in the HCVcc model. To our knowledge, this is the first report regarding RFs in non-infected and HCV-infected Huh-7.5 cells. In addition, because Huh-7.5 are human hepatoma cells, our data complement the existing evidence for RF accumulation in cancer [5, 62–64].

Our analyses demonstrated that virtually all classes of cellular RNA reproducibly generated stable RFs in Huh-7.5 cells. In accordance with findings from earlier reports [3, 65, 66], our data suggest that the biogenesis of RFs is not a random process. This opinion is supported at least by two facts. First of all, we observed that in all groups of RFs, except rRFs, individual RNA species clustered around two or more length ranges. Such a length distribution obviously distinguishes RFs from other well-known small RNAs, such as miRNA, siRNA and piRNA, which have been shown to cluster around a single length range. In contrast to other RF groups, rRFs – and consequently the process of rRNA fragmentation – did not display any features of specificity. The length distribution of rRNAs suggests that they are the products of processive digestion by cellular exonucleases. This cleavage pattern could be attributed to stress conditions in hepatoma cells. Interestingly, the additional stress induced by HCV infection further increased rRNA fragmentation. Analogous increases in rRF accumulation have been previously reported in response to RSV [31] and ASGV infections [34].

Our analyses also revealed that some regions of parental RNAs preferentially give rise to RFs. Among the tRFs, those with retained 5′ ends, especially tRF-5A, were the most numerous in the HCVcc model. However, another class of tRNA-derivatives, termed tRFΔ, was likewise considerable. Thus far, only a few reports have described internal tRFs [10, 63, 67, 68], and only one of them depicted this class as rich and potentially significant [67]. Our results strongly support this previous observation because as much as 25% of all tRFs lacked both ends of the mature tRNA. Notably, we found that in the case of tRFΔ, cleavage sites frequently localized to the originally double-stranded regions. This is a novel finding because loops were previously indicated as the primary starting positions of internal tRFs [67]. Based on the distribution of cleavage sites and the assumption that single-stranded regions are more prone to cleavage, we propose two scenarios of tRFΔ biogenesis. The first step, the primary cleavage of tRNA within a single-stranded region, is common for both scenarios, and the next step/s associated with the secondary cleavage is/are different. According to the first scenario, after the primary cleavage, the tRNA fragments remain base-paired and the overall fold of the molecule is retained. The secondary cleavage occurs in a non-base-paired region; thus, both ends of the generated species originate within single-stranded regions of the full-length tRNA. According to the second scenario, after the primary cleavage, the tRNA fragments disassociate and undergo structural rearrangements that lead to the stem unfolding. The secondary cleavage also occurs within a single-stranded region of the resultant tRF. Consequently, one end of the generated tRFΔ is located in the single-stranded and the other in the single- or double-stranded regions of the original tRNA (single-stranded region present in the rearranged molecule can form single- or double-stranded structure in the original molecule before rearrangement). We believe that structural rearrangements are more plausible than the involvement of double-stranded RNA (dsRNA)-specific ribonucleases in the secondary cleavage. If a dsRNA-specific ribonuclease participated in the digestion of the tRNA stems, such cleavages would be more frequently observed in cases of species with one of the ends retained.

In view of the fact that the vast majority of tRNAs are complexed with proteins [69, 70], it has remained unclear when these molecules are cleaved and whether the two portions of the nicked tRNA dissociate [71]. Previously, it has been shown that the extent of cleavage is higher upon active translation. During this process, tRNAs are highly likely to exist in an unbound form because they are frequently relocated from a complex with EF1A to a complex with aminoacyl-tRNA synthetase. This observation suggests that free tRNA can be the preferred cleavage substrate [70]. In turn, our data provide an indication that the dissociation of tRNA fragments does occur, at least in a fraction of the nicked tRNAs. Another factor influencing the biogenesis of tRFs might be differences in the affinity for protein binding observed for full-length and truncated tRNAs. For example, full-length tRNA, but not tRFs, have been shown to bind with the translation factors eIF2α and EF1A [70], whereas tRFs, but not tRNA, preferentially bind with cytochrome c upon stress [69]. Clearly, interactions with proteins can affect the secondary and tertiary structure of RNA and, consequently, can decide which regions are exposed and which are protected from cleavage. Interestingly, our results suggest that a fraction of snRFs (cleaved from both ends of the parental molecules) and YRFs can also be formed via a mechanism that involves a structural rearrangement step. Because these groups of fragments were less numerous in the present study, future studies are required to validate the relevance of this observation.

We also found that a number of snoRNA-, snRNA- and Y RNA-derived RFs included regions relevant for the function of their parental molecules. Most snoRFs carried the conserved boxes (D, C and/or D′), which is consistent with previously published data [66, 72]. The conserved boxes are essential for the interactions between snoRNA and their protein partners and thus for the formation of functional snoRNP complexes capable of modifying the target RNAs. In the case of snRFs, 70% of the fragments contained motifs engaged in interactions with spliceosomal Sm proteins, whereas none of them had any of the more exposed segments involved in binding with pre-mRNA. Most fragments derived from Y RNA included one strand of the Ro60 binding region and/or one strand of the segment essential for DNA replication. Both of these regions recruit protein partners, Ro60 and DNA replication initiation proteins, respectively [58]. The presence of the conserved protein-binding motifs in the identified RFs suggests that proteins interacting with their parental molecules are important factors that affect the formation and maintenance of snoRNA-, snRNA- and Y RNA-derived RFs. All of these proteins can function as shields that protect RNA or their fragments from nucleases. At the same time, this indicates that RFs can interfere with the functioning of their parental molecules by sequestering their protein partners.

In contrast to earlier reports [66, 72], we identified a considerable proportion of snoRFs that included the entire guide sequence (approximately 41%). Guide sequence hybridizes to the target rRNA to ensure its site-specific modification [73]. Generation of such fragments would possibly involve cleavages within the protein-shielded regions. These cleavage sites should be surveyed in detail in the context of the formation of particular snoRNP, which is a multistep process involving RNA structural rearrangements and the sequential recruitment of proteins [74]. The outcomes of such analysis are likely to indicate the points at which cleavages are feasible and thus provide insight into the biogenesis of snoRFs.

The results presented here suggest that RNA structure and the RNA-protein interactome are the major factors shaping RF structure and composition. Interestingly, small non-coding RNAs detected in chloroplasts have been proposed to be footprints of pentatricopeptide repeat proteins [75]. In addition, protection by tRNA-binding proteins has been demonstrated to counteract the degradation of hypomodified tRNA in yeast when polymerase III transcription is repressed [76]. Further studies are required to unravel the extent to which protein-mediated nuclease protection takes part in the generation of particular RF groups. However, one can speculate that the biogenesis of RFs involves multiple mechanisms, from directed cleavage by specific nucleases (some of which have already been identified [1]) to protection by RNA-binding proteins. The engagement of multiple mechanisms in the RF production is supported by several lines of evidence. Firstly, unlike miRNA, siRNA and piRNA, RFs show two or more peaks in their length distribution plots. Secondly, we identified three major patterns of parental RNA cleavage into RFs. Based on our observations, one can conclude that in the exploration of RF biogenesis the survey of RNA-binding proteins appears equally important as the search for specific nucleases.

Recently Selitsky and coworkers have characterized the tRF abundance in the human liver [35]. To this end, they measured the total amount of all tRFs and compared it to the cumulative level of all miRNAs. As a result, tRFs were found to be abundant in non-malignant liver tissue and to be increased in cases of chronic viral hepatitis (B or C) to levels that surpass that of miRNAs. In hepatocellular carcinoma (HCC) tissues of HBV- or HCV-infected patients, the accumulation of tRFs was shown to be reduced [35]. We did not observe a global predominance of tRFs over miRNA. However, we calculated the relative abundances of representative *tRF* species instead of only comparing the proportions of reads that map to particular RNA classes. In this way we were able to observe that most miRNAs accumulate to levels several orders of magnitude lower than that of *miR-122*. Similar patterns of relative accumulation were revealed for *tRFs*, *snoRFs*, *YRFs* and, to some extent, for *snRFs* as well. Based on the estimation that miR-122 is present at approximately 15,000 copies per cultured hepatoma cell [77] and that as little as 4 copies per cell are enough for a regulatory RNA to exert its effect [78], one can expect the identified *RFs* to have functional impacts. Importantly, we discovered that not only *tRFs* but also the derivatives of other RNAs accumulated to levels comparable with the abundance of individual miRNAs. Selitsky and coworkers showed that derivatives of tRNA-Val and tRNA-Gly were the 2 most abundant tRFs in livers of patients with chronic hepatitis C (CHC). Their levels were increased upon infection compared to non-infected livers (i.e., they exceeded the amount of miR-122) but became reduced again in cancerous tissues [35]. We identified these *tRFs* as being among the most abundant in Huh-7.5 cells; however, their levels were not higher than that of *miR-122* and were not elevated upon HCV infection. These discrepancies can be attributed to the fact that Huh-7.5 cells are hepatoma cells and thus display the characteristics of cancerous tissue. In addition, it is plausible that the RF levels differ between the beginning of the infection (observed in the HCVcc model) and a prolonged exposure to HCV during CHC. Nevertheless, the existence of the same tRFs in Huh-7.5 cells and in liver tissues of CHC patients supports the physiological relevance of the HCVcc model for studies of non-coding RNA. This model could be applied to investigate the potential impact of tRNA-Val and tRNA-Gly fragments on the course of HCV infection.

Interestingly, among the 25 most abundant *RFs*, we identified an 18-mer derived from a short RNA previously found to interact with tRNAseZ^L^ [20]. Although the function of this particular short RNA was not examined, the same report revealed that tRNAseZ^L^ used other fragments of tRNA and rRNA as guide molecules to target mRNA of, respectively, PPM1F (protein phosphatase Mg^2+^/Mn^2+^ dependent 1F) and DYNC1H1 (dynein cytoplasmic 1 heavy chain 1). Furthermore, tRNAseZ^L^ was demonstrated to be ubiquitous in diverse cell types, including HepG2 hepatoma cells, and to be involved in the regulation of apoptosis [20]. In this context, the abundant 18-mer identified in Huh-7.5 cells represents an interesting candidate for future studies of its functional relevance. The most abundant *RFs* identified in Huh-7.5 cells included also several derivatives of snoRNA. The fragments originating from SNORD44 and SNORD78 overlapped with the species recently shown to be up-regulated in malignant prostate tissue (they were identical or differed by several terminal nucleotides). Interestingly, a derivative of SNORD78 highly similar to the one identified in Huh-7.5 cells has been proposed as a novel prognostic biomarker of metastatic disease in prostate cancer [79]. The last of the 25 most abundant *RFs* in Huh-7.5 cells, which was also significantly up-regulated upon HCV infection at 96 hpi, was a derivative of hY4. A small RNA of the same sequence (termed ASR2) was discovered in cells infected with Eppstein-Barr virus (EBV), where it was up-regulated during the lytic phase of the viral replication cycle [80]. Furthermore, ASR2 was shown to specifically bind with Ago1 (and not other Ago types) thereby being stabilized at a relatively high level (depletion of Ago1 resulted in a marked decrease in ASR2 content). In addition, this small RNA mediated the silencing of its target mRNA by binding within the mRNA’s 3′ UTR [80]. Our results encourage functional studies of the hy4 derivative in the context of HCV infection.

Our analyses revealed that HCV infection in a cell culture model generally did not impact the highly abundant *RFs* but instead elevated the accumulation of the low copy number fragments. It remains to be established to what extent this minor influence of HCV on the global RF pool is conditioned by the background of Huh-7.5 cells, which are already rich in RFs before the onset of an infection. Although increased amounts of RFs have been generally associated with cancer [5, 62, 64, 81], the levels of their accumulation have been shown to be cell-specific [6, 66]. In addition, viral infections have been demonstrated to trigger the increased production of RFs [31, 34]; however, this is not a general rule [33].

In the group of differentially accumulated *RFs,* we identified several candidates that reached considerable levels and whose role in HCV infection is worth experimental evaluation (Table 2). We observed a dramatic increase in the cleavage of snRNA (primarily U1) in infected cells, which resulted in the accumulation of some *snRFs* reaching up to about 7% of the amount of *miR-122* (Table 3). The data regarding snRNA fragmentation are limited, and it currently remains unknown whether the observed cleavage and its products might be functionally relevant. Interestingly, circulating U2 snRNA fragments have been described as diagnostic biomarkers in several types of cancer [82–86].

## Conclusions

Here, we provide for the first time an overall picture of the RF accumulation in the HCVcc model. We found that virtually all RNA classes are sources of RFs, and we explored the cleavage patterns across several constitutive non-coding RNA classes, which provides novel insight into RF biogenesis and its potential significance. Although HCV infection did not induce profound quantitative or qualitative changes in the profile of RF accumulation, our data indicate that this rich and diversified population of small RNAs should be considered as a significant component of the infection environment. Accordingly, we identified a number of candidate RFs that potentially can be implicated in the course of HCV infection and, consequently, deserve further experimental study. We believe that our results will make a valuable contribution to the previous characterization of the transcriptomic, miRNomic, proteomic and metabolomic landscapes of the HCVcc model [51–53].

## Methods

### Cell culture and viral infection

The plasmid encoding the genome of the JFH-1 HCV strain (genotype 2a) was kindly provided by T. Wakita and was used to generate high-titer stocks of cell culture-produced virus (HCVcc) according to a previously published procedure [87]. The human hepatoma cells (Huh-7.5), kindly provided by C. Rice, were cultured as previously described [88] and inoculated with a viral stock of 46,400 TCID50/ml, at a multiplicity of infection (moi) of 1 or 0.1, for 2 h at 37 °C. Subsequently cells were washed and cultured for 72 h (for a moi of 1) or 96 h (for a moi of 0.1) when approximately 80% of cells were infected. Percentages of infected cells were estimated via the detection of HCV core proteins using a previously described immunofluorescence assay [89]. The infected cells were fixed with 4% paraformaldehyde (PFA), permeabilized with 0.5% Triton X-100 and blocked with a buffer containing 1% gelatin and 0.1% Tween 20 in PBS. Each step was followed by a wash with PBS. Next, cells were incubated for 2 h with mouse monoclonal anti-HCV core antibodies (ACAP27, Bio-Rad), washed with 0.1% Tween 20 in PBS and incubated for 1 h with Alexa Fluor 568-labeled goat anti-mouse IgG H + L (Invitrogen). Finally, cells were washed, stained with 4′,6-diamidino-2-phenylindole (DAPI) in Vectashield medium (Vector Laboratories) and examined under a Zeiss Widefield ApoTome AxioCam upright microscope. The percentage of infected cells (expressed as the average number of infected cells per 100 cells) was determined in multiple random fields of view. All experiments were performed in duplicate.

### RNA isolation and quality control

RNA was isolated from infected and control Huh-7.5 cells using a *mir*Vana miRNA Isolation Kit (Ambion) according to manufacturer’s instructions. For each of the two biological replicates, the four following RNA samples were obtained: from infected cells collected at 72 h post inoculation (hpi) (72I), from infected cells collected at 96 hpi (96I) and from the corresponding controls (72C and 96C, respectively). A two-step isolation method was used, where the first step was the extraction of total RNA, and the second the fractionation of the total RNA into short (< 200 nt) and long (> 200 nt) RNA. Next, the integrity and purity of the RNA samples were assessed using a NanoDrop 2000 spectrophotometer (Thermo Scientific) and Bioanalyzer 2100 (Agilent) according to the manufacturers’ instructions. The samples were analyzed with a RNA 6000 Nano Assay. The representative electropherograms obtained for the sequenced samples (for total, long and short RNA pools) are shown in Additional file 1: Figure S1. In addition, the RNA integrity number (RIN) was determined for the total and long RNA pools. We collected short RNA fractions for further study only from those samples for which the RINs obtained for both total and long RNA exceeded nine.

### Library construction and sequencing

Each short RNA sample from one biological replicate (72I, 72C, 96I and 96C) was used to obtain the NGS library. For this purpose, the TruSeq Small RNA kit (Illumina) was used according to the manufacturer’s instructions. Briefly, 0.5 μg of the short RNA fraction was subjected to indexed adapter ligation and reverse transcription, followed by library amplification (11 cycles). The amplified cDNA was quantified on a NanoDrop 2000 spectrophotometer (Thermo Scientific), and the product length was analyzed with a Bioanalyzer 2100 and a High Sensitivity DNA Assay (Agilent). The four samples had similar DNA concentrations (222–264 ng/μl) and DNA peak profiles. To ensure uniform size fractionation, the indexed libraries were combined and size-separated in 6% denaturing polyacrylamide gel (Novex). The fraction located between the tRNA band and adapter dimer band was collected and recovered from the gel. The effectiveness of the size selection procedure was confirmed via a High Sensitivity DNA Assay (Agilent). The entire procedure was then repeated for the second set of 72I, 72C 96I, and 96C short RNA samples. Each of the gel-eluted mixtures of the four libraries (without DNA precipitation) was sequenced independently on an Illumina Genetic Analyzer IIx for 100 cycles.

### NGS data analysis

The raw sequencing reads were assigned to the appropriate libraries (i.e., 72I, 72C, 96I and 96C) based on their indexes and checked for quality with Prinseq [90], and the adapter sequences were removed with TagCleaner [91]. The adapter removal step is very important, as we analyzed further only the reads that carried at least the whole adapter sequence. The detection of the adapter proved that a particular read indeed represented an authentic sequence of the entire small RNA and not a partial sequence of a longer RNA molecule. Next, to ensure that the analyzed reads were of high quality, we removed those with at least one nucleotide that had a Phred quality score below 30. Then, reads were filtered to discard sequences shorter than 15 nt. In the next step, only RNA species represented by more than 15 reads per million in at least two corresponding samples (biological replicates) were selected. Those that did not meet this criterion were excluded from further analysis to omit negligible molecules. Finally, the individual RNA species were sequentially mapped with Bowtie [92] to miRbase [93], fRNAdb [94], the HCV JFH-1 genome [95] and the human genome (hg19) [96], allowing multiple mapping. This mapping was performed in an iterative manner. In the first round, no mismatches were allowed. All reads that mapped with 0 mismatches to either miRbase, fRNAdb, HCV JFH-1 or hg19 were annotated, and the rest was subjected to further rounds of mapping with an increasing number of mismatches allowed (1, 2 and 3). Read counts were normalized based on the library size using the edgeR Bioconductor package [97].

### Differential accumulation analysis

A differential accumulation analysis was performed for representative RNA species, which were selected according to the following procedure. The sequences of all RFs derived from the same parental full-length RNA were aligned and compared. Next, in each of the obtained alignments, the RF with the highest mean abundance across all samples (normalized read count) was selected to initiate a cluster. Subsequently, all RFs that mapped within the same region of parental RNA and differed in length from the first one by +/− 10% were added to the cluster. If not all RFs were clustered, the next RF with the highest mean abundance among the remaining RNA species was selected to initiate the second cluster, to which suitable RFs were subsequently added. The procedure was repeated until no RFs remained ungrouped. To ensure appropriate clustering, the initial segregation results were checked. It was revealed that some RFs, because of their alignment position and length, could fit into more than one cluster. In such cases, for each of the considered clusters to which a particular RF could fit, the mean length of the remaining cluster members was calculated. The RF was assigned to the cluster for which the difference between mean length and RF length was the smallest. Finally, the representative RF for each cluster was determined. For this, we first added the mean abundances of all cluster members to obtain a total value for a cluster. Then, we calculated the summarized mean abundance for each nucleotide covered by cluster members in the parental RNA sequence. The representative RF was the one with a summarized mean abundance for each nucleotide that was not less than 40% of the total value for the cluster. In this way, we ensured that clusters were represented by the most distinctive RFs. The clustering operation reduced the initial library of RNA species. Consequently, from this stage on, the initially obtained normalized read counts could not be further considered. Therefore, the abundance of each representative RF was calculated again by adding its raw read count to the raw read counts of other RFs belonging to the same cluster. These values were then normalized by the reduced library size. Sequences of individual RNAs that mapped to a certain miRNA were assigned to one cluster. The reference sequences from miRbase were taken as representative sequences, and their abundance was calculated as described for representative RFs. In total, 1001 clusters were obtained (827 for RFs and 174 for miRNA), each represented by one RNA species and a newly calculated abundance. Subsequently, a differential accumulation analysis was performed for the representatives using edgeR and comparing infected cells to controls. We considered representative RNAs with log_2_ fold change (log_2_FC) ≥ 2 or ≤ −2 and a false discovery rate (FDR) < 0.05 as being significantly differentially accumulated.

## Additional file

Additional file 1: Figure S1. Representative electropherograms for total RNA, long RNA (RNA > 200 nt) and short RNA (RNA < 200) samples (Agilent Bioanalyzer 2100, RNA 6000 Nano Assay). Short RNA fractions were taken for sequencing. To ensure the highest quality, only those samples were selected, for which both corresponding total and long RNA RINs exceeded 9. **Figure S2.** 2D–PAGE analysis of short RNA fractions isolated from non-infected (72C, 96C) and infected (72I, 96I) Huh-7.5 cells. For each sample representative results from three replicates are shown. **Figure S3.** (A) Contribution of individual species representing particular groups (miRNA and RNA fragments) to the total number of all RNA species identified in this study. (B) Contribution of the accumulation of individual species representing particular groups (miRNA and RNA fragments) to the total normalized read count of all RNA species identified in non-infected (72C, 96C) and infected (72I, 96I) Huh-7.5 cells. **Figure S4.** Schematic representation of tRNA secondary structure. **Figure S5.** Simplified scheme of C/D box and H/ACA box snoRNA secondary structures. Rectangles indicate the functionally relevant regions. **Figure S6.** Schematic representation of snRNA secondary structures. Predicted secondary structures of snRNA depicted in black are based on: Patel, A. A. and Steitz J. A. (2003) Splicing double: insights from the second spliceosome. *Nat Rev. Mol Cell Biol*, 4, 960–970. Rectangles indicate the functionally relevant regions. U6 snRNA and U6atac snRNA depicted in gray are simplified and do not include the predicted motifs of secondary structures. **Figure S7.** Schematic representation of Y RNA secondary structures. Predicted secondary structures of Y RNA are based on: Kowalski, M.P. and Krude, T. (2015) Functional roles of non-coding Y RNAs. *Int J Biochem Cell Biol*, 66, 20–29. Rectangles indicate the functionally relevant regions. (PDF 1262 kb)

## Abbreviations

2D–PAGE: Two dimensional polyacrylamide gel electrophoresis
APOER2: Apolipoprotein E receptor 2
ASGV: Apple stem grooving virus
BLAST: Basic local alignment search tool
CHC: Chronic hepatitis C
DYNC1H1: Dynein cytoplasmic 1 heavy chain 1
EBV: Eppstein-Barr virus
EF1A: Eukaryotic translation elongation factor 1 alpha
eIF2α: Eukaryotic initiation factor 2 alpha
FGF-2: Fibroblast growth factor-2
HCC: Hepatocellular carcinoma
HCV: Hepatitis C virus
HCVcc: HCV cell culture
hMPV: Human metapneumovirus
hpi: Hours post inoculation
IRES: Internal ribosome entry site
miRNA: Micro RNA
mRNA: Messenger RNA
NGS: Next generation sequencing
piRNA: Piwi-interacting RNA
PPM1F: Protein phosphatase Mg2+/Mn2+ dependent 1F
RF: RNA fragment
RIN: RNA integrity number
RISC: RNA-induced silencing complex
rRNA: Ribosomal RNA
RSV: Respiratory syncytial virus
siRNA: Small interfering RNA
SL: Stem-loop
snoRNA: Small nucleolar RNA
snRNA: Small nuclear RNA
TLR3: Toll-like receptor 3
tRNA: Transfer RNA
