# Supplementary material for: Small RNA fragments derived from multiple RNA classes – the missing element of multi-omics characteristics of the hepatitis C virus cell culture model
Source: BMC Genomics. 2017 Jun 30;18:502. doi: 10.1186/s12864-017-3891-3 (PMC5493846; doi:10.1186/s12864-017-3891-3)
Supplement: Additional file 1: Figure S1. — Representative electropherograms for total RNA, long RNA (RNA > 200 nt) and short RNA (RNA < 200) samples (Agilent Bioanalyzer 2100, RNA 6000 Nano Assay). Short RNA fractions were taken for sequencing. To ensure the highest quality, only those samples were selected, for which both corresponding total and long RNA RINs exceeded 9. Figure S2. 2D–PAGE analysis of short RNA fractions isolated from non-infected (72C, 96C) and infected (72I, 96I) Huh-7.5 cells. For each sample representative results from three replicates are shown. Figure S3. (A) Contribution of individual species representing particular groups (miRNA and RNA fragments) to the total number of all RNA species identified in this study. (B) Contribution of the accumulation of individual species representing particular groups (miRNA and RNA fragments) to the total normalized read count of all RNA species identified in non-infected (72C, 96C) and infected (72I, 96I) Huh-7.5 cells. Figure S4. Schematic representation of tRNA secondary structure. Figure S5. Simplified scheme of C/D box and H/ACA box snoRNA secondary structures. Rectangles indicate the functionally relevant regions. Figure S6. Schematic representation of snRNA secondary structures. Predicted secondary structures of snRNA depicted in black are based on: Patel, A. A. and Steitz J. A. (2003) Splicing double: insights from the second spliceosome. Nat Rev. Mol Cell Biol, 4, 960–970. Rectangles indicate the functionally relevant regions. U6 snRNA and U6atac snRNA depicted in gray are simplified and do not include the predicted motifs of secondary structures. Figure S7. Schematic representation of Y RNA secondary structures. Predicted secondary structures of Y RNA are based on: Kowalski, M.P. and Krude, T. (2015) Functional roles of non-coding Y RNAs. Int J Biochem Cell Biol, 66, 20–29. Rectangles indicate the functionally relevant regions. (PDF 1262 kb) [file 12864_2017_3891_MOESM1_ESM.pdf]

Small RNA fragments derived from multiple RNA classes – the missing element of multi-omics characteristics of the hepatitis C virus cell culture model

Paulina Jackowiak[1], Anna Hojka-Osinska[1], Anna Philips[1], Agnieszka Zmienko[1,2], Lucyna Budzko[1], Patrick Maillard[3], Agata Budkowska[3*], Marek Figlerowicz[1,2**]

[1] Institute of Bioorganic Chemistry, Polish Academy of Sciences, Noskowskiego 12/14, 61-704 Poznan, Poland

[2] Institute of Computing Science, Poznan University of Technology, Piotrowo 3A, 60-965 Poznan, Poland

[3] Institut Pasteur, Hepacivirus and Innate Immunity, CNRS, UMR3569, 75724 Paris, France

keywords: non-coding RNA, RNA fragments, tRF, HCV

* at present: Scientific Advisor for the Department of International Affairs, Institut Pasteur, 75724 Paris, France

** Corresponding author
Prof. Marek Figlerowicz
Institute of Bioorganic Chemistry
Polish Academy of Sciences
Noskowskiego 12/14
61-704 Poznan, Poland
e-mail: marekf@ibch.poznan.pl

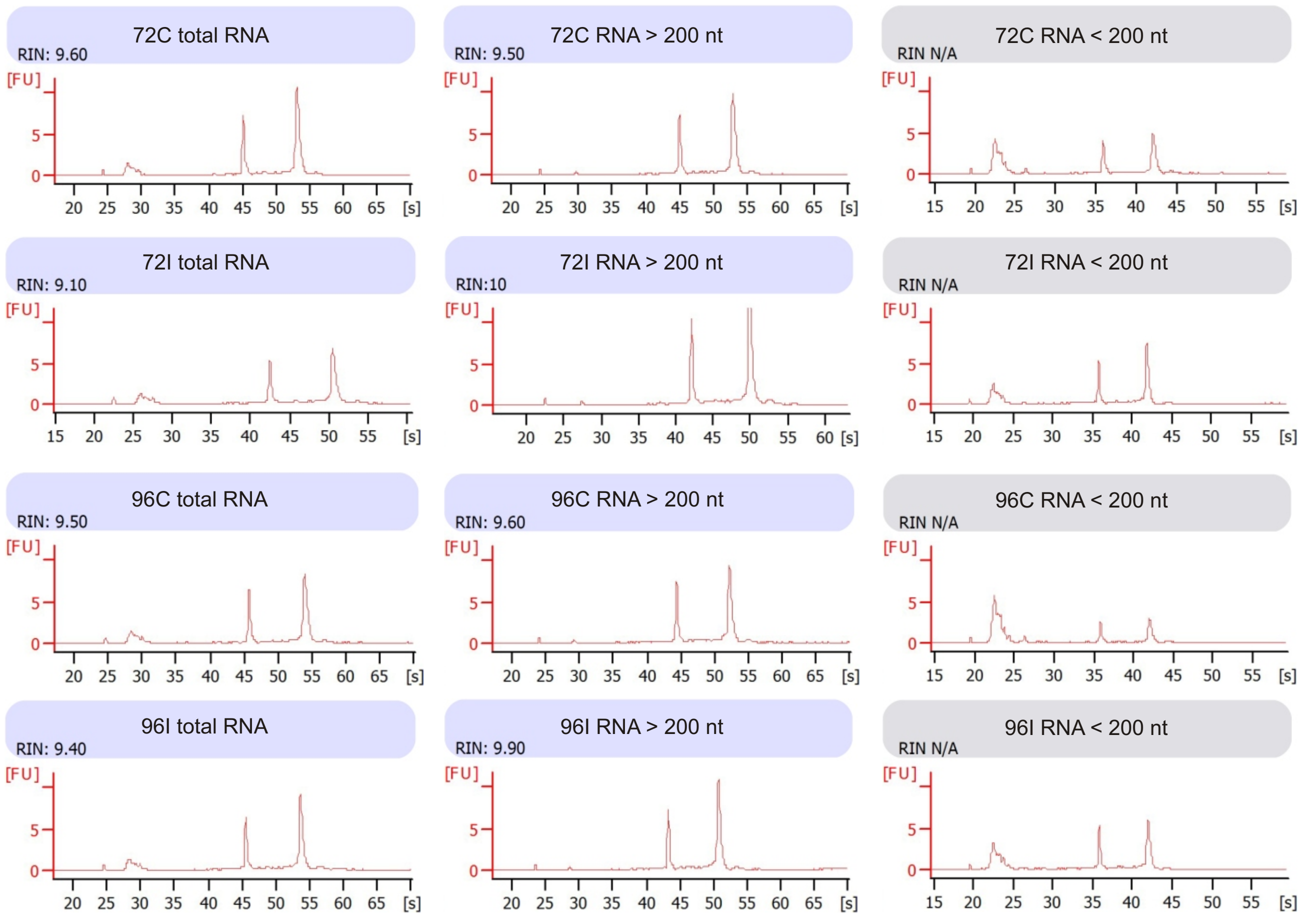

Supplementary Figure 1. Representative electropherograms for total RNA, long RNA (RNA>200nt) and short RNA (RNA<200) samples (Agilent Bioanalyzer 2100, RNA 6000 Nano Assay). Short RNA fractions were taken for sequencing. To ensure the highest quality, only those samples were selected, for which both corresponding total and long RNA RINs exceeded 9.

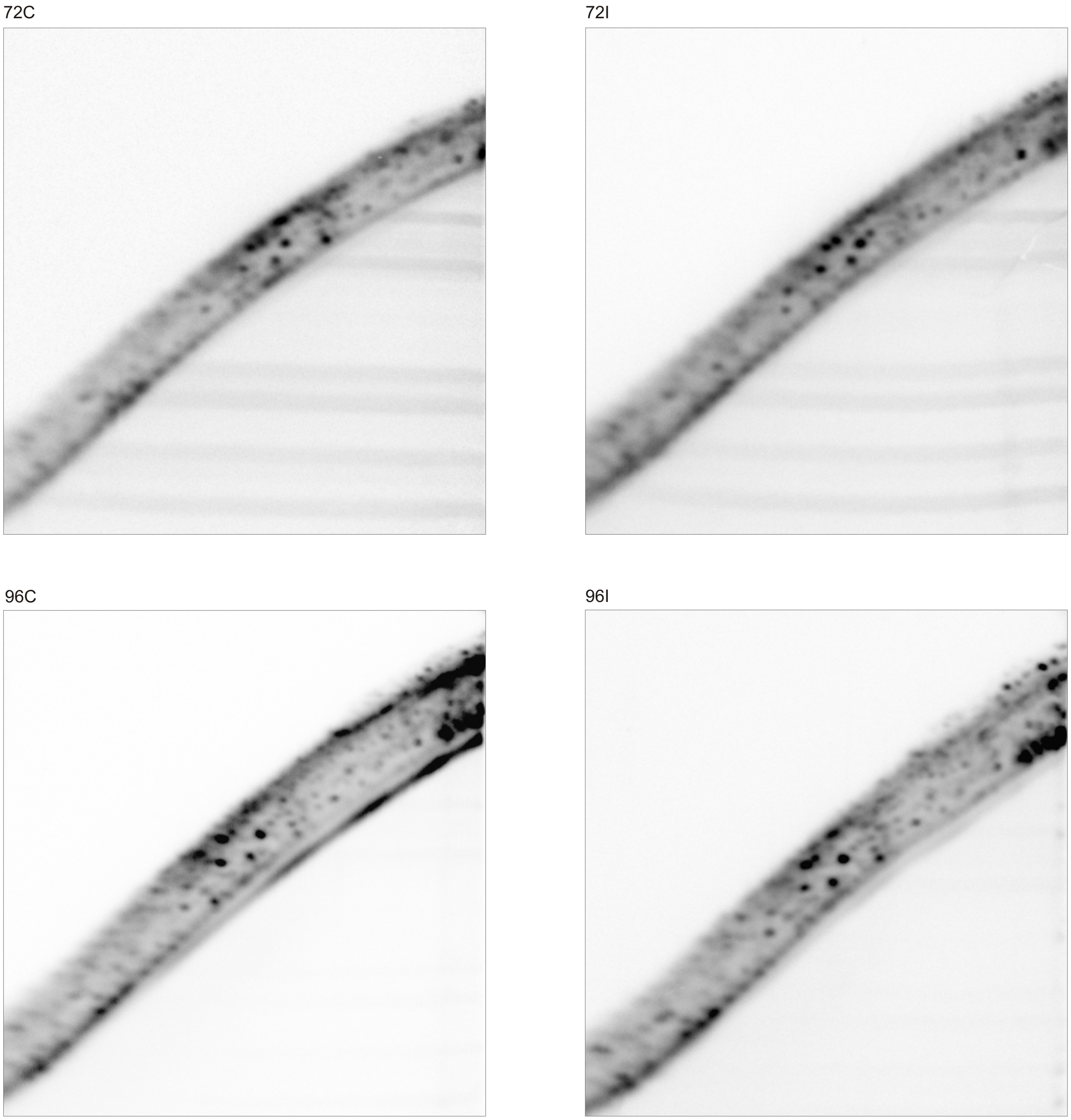

Supplementary Figure 2. 2D-PAGE analysis of short RNA fractions isolated from non-infected (72C, 96C) and infected (72I, 96I) Huh-7.5 cells. For each sample representative results from three replicates are shown.

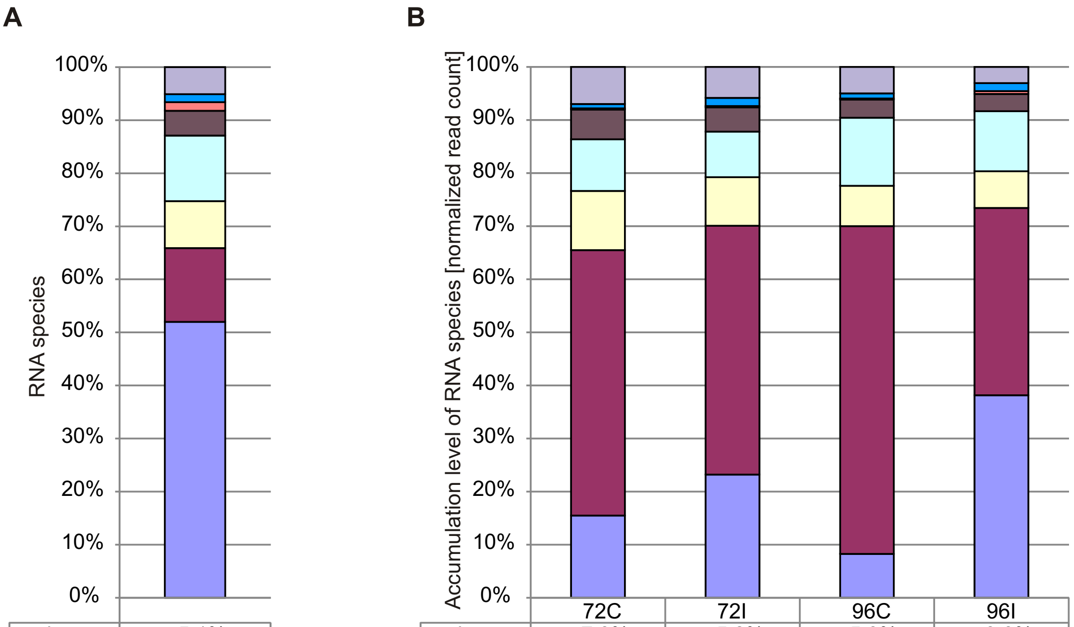

A

other	5.1%
Y RNA	1.5%
snRNA	1.6%
mRNA	4.7%
tRNA	12.4%
snoRNA	8.8%
miRNA	13.9%
rRNA	52.0%

B

	72C	72I	96C	96I
other	7.0%	5.8%	5.0%	3.0%
Y RNA	0.8%	1.5%	1.0%	1.5%
snRNA	0.2%	0.2%	0.2%	0.5%
mRNA	5.6%	4.6%	3.4%	3.2%
tRNA	9.7%	8.6%	12.8%	11.3%
snoRNA	11.2%	9.2%	7.6%	6.9%
miRNA	50.0%	46.9%	61.8%	35.3%
rRNA	15.5%	23.2%	8.3%	38.2%

Supplementary Figure 3. (A) Contribution of individual species representing particular groups (miRNA and RNA fragments) to the total number of all RNA species identified in this study. (B) Contribution of the accumulation of individual species representing particular groups (miRNA and RNA fragments) to the total normalized read count of all RNA species identified in non-infected (72C, 96C) and infected (72I, 96I) Huh-7.5 cells.

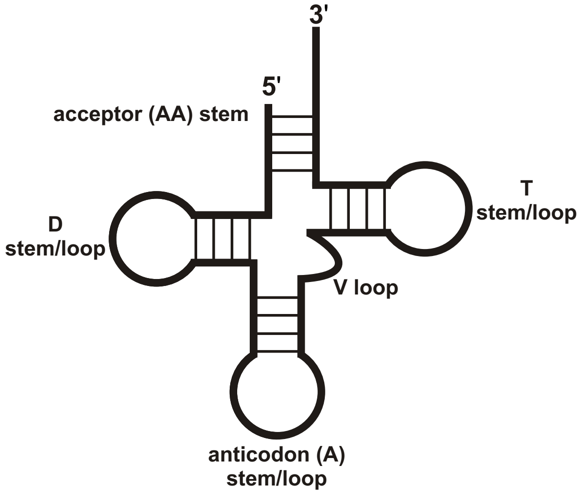

Supplementary Figure 4. Schematic representation of tRNA secondary structure.

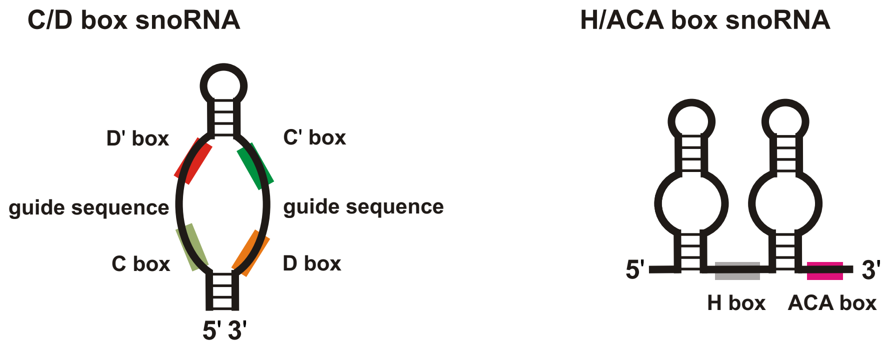

Supplementary Figure 5. Simplified scheme of C/D box and H/ACA box snoRNA secondary structures. Rectangles indicate the functionally relevant regions.

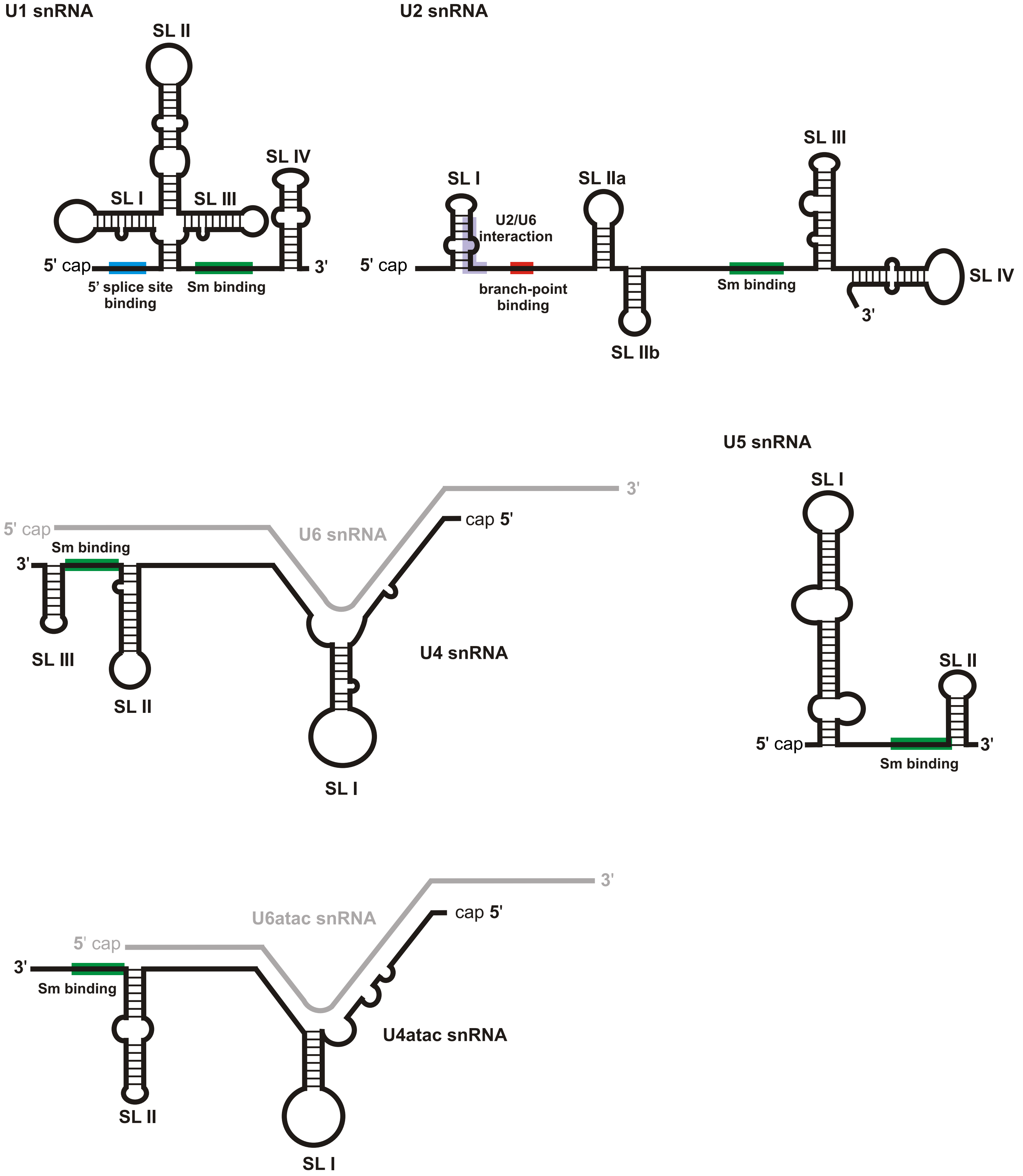

Supplementary Figure 6. Schematic representation of snRNA secondary structures. Predicted secondary structures of snRNA depicted in black are based on: Patel, A. A. and Steitz J. A. (2003) Splicing double: insights from the second spliceosome. *Nat Rev Mol Cell Biol*, 4, 960-970. Rectangles indicate the functionally relevant regions. U6 snRNA and U6atac snRNA depicted in gray are simplified and do not include the predicted motifs of secondary structures.

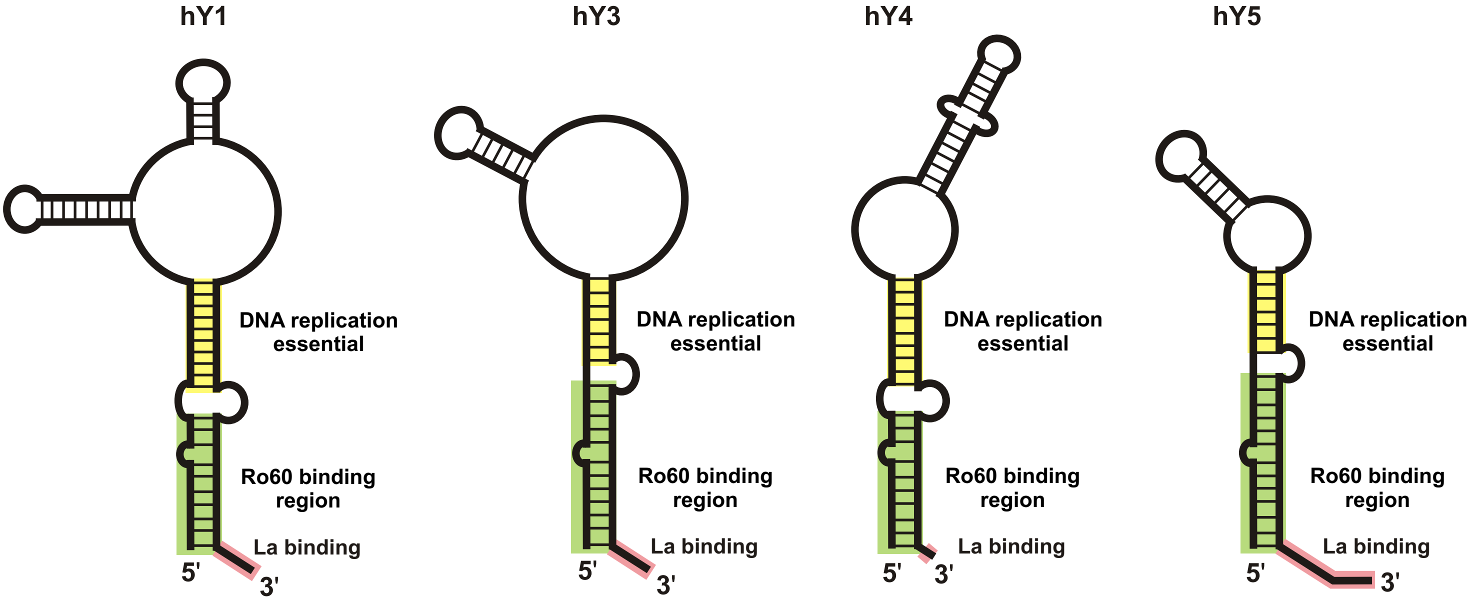

Supplementary Figure 7. Schematic representation of Y RNA secondary structures. Predicted secondary structures of Y RNA are based on: Kowalski, M.P. and Krude, T. (2015) Functional roles of non-coding Y RNAs. *Int J Biochem Cell Biol*, 66, 20-29. Rectangles indicate the functionally relevant regions.
